# Safety and efficacy of antigen-specific therapeutic approaches for multiple sclerosis: Systematic review

**DOI:** 10.1371/journal.pone.0320814

**Published:** 2025-05-19

**Authors:** Hatice Kübra Öztürk, Ondřej Slanař, Danica Michaličková

**Affiliations:** Institute of Pharmacology, First Faculty of Medicine and General University Hospital, Charles University, Prague, Czech Republic; University of Rijeka Faculty of Medicine: Sveuciliste u Rijeci Medicinski fakultet, CROATIA

## Abstract

**Introduction:**

The objective of this systematic review is to evaluate the efficacy and safety of antigen-specific tolerance-inducing therapeutic approaches (products based on peptides, DNA and T cells) versus placebo or other comparators, where possible, in adult multiple sclerosis (MS) patients.

**Methods:**

PubMed, CINAHL, Web of Science, Cochrane and International Clinical Trials Registry Platform, ClinicalTrials.gov were searched for published and unpublished studies. Screening, critical appraisal, and data extraction for included studies were carried out by two independent reviewers. For efficacy, phase I, II and III clinical trials (randomized/non-randomized; double blind/single blind/unblinded; single-center/multicenter; single-arm/two-arm) and for safety, phase I, II and III clinical trials (randomized/non-randomized; double blind/single blind/unblinded; controlled/uncontrolled; single-center/multicenter; single-arm/two-arm) were included. Observational studies (cross-sectional studies, cohort studies, case studies/reports etc), review articles, systematic reviews, meta-analysis, preclinical and pilot studies were excluded. All included studies were critically appraised using standardized JBI tools, with no exclusions based on methodological quality. Where possible, studies were pooled in statistical meta-analysis, presented in tabular format, and accompanied by narrative synthesis. The Grading of Recommendations, Assessment, Development and Evaluation (GRADE) approach for grading the certainty of evidence.

**Results:**

Search yielded 2644 results and in total 26 studies were included in the final analysis. Twelve studies were RCTs and 14 were quasi-experimental. In total, there were 1427 subjects from the RCTs, and 314 from non-RCTs. Sample size of studies ranged from 10 to 612 patients. All studies included adult patients, principally aged 18–55/65 years. Critical appraisal scores for the RCTs were in the range 31% to 92%. For quasi-experimental studies, critical appraisal scores were in the range 45% to 78%. Due to high heterogeneity of the studies, efficacy of all antigen-specific treatment remained ambiguous. For all three types of treatment, there was no statistical difference in occurrence of adverse effects (AEs) between the treatment- and placebo-related AEs (for DNA-based treatment RR was 1.06 (0.94–1.10), p = 0.334; for peptides-base treatments RR was 1.04 (0.90–1.08), p = 0.115; for T-cells-based treatments RR was 1.31 (0.97–1.76), p = 0.08). There were no differences in RR for serious AEs (SAEs) between the treatments either for DNA-based treatment (RR was 0.63 (0.25–1.58), p = 0.322) or peptide-based treatment (RR was 0.86 (0.62–1.19), p = 0.361). There were no reported SAEs for T cell-based treatments, so meta-analysis for these therapies was not performed. The most frequent AEs were local reactions to injection, such as redness, erythema, pain.

**Discussion:**

Antigen-specific tolerance-inducing therapeutic approaches appeared to be safe. However, the certainty for these results was very low for SAEs in peptide- and DNA-based therapies, whereas it was low for AEs in DNA- and T cells-based therapies and moderate for AEs in peptide-based therapies. The efficacy of antigen-specific therapies remains ambiguous. Larger, well-designed studies with high level quality are needed to ensure ultimate conclusions.

**Registration:**

The registration number provided following registration of the protocol in PROSPERO is ‘CRD42021236776’.

## Introduction

Multiple sclerosis (MS) is an inflammatory and neurodegenerative disease of the central nervous system (CNS) that is a common cause of serious physical non-traumatic disability in young adults [1,2]. The incidence of the disease is twice as high in women as in men and it mostly affects young adults with the average age of diagnosis being 32. The prevalence of MS has increased since 2013. Currently it is estimated that 2.8 million people suffer from MS worldwide [1,2]. Patients may experience one or more of the following symptoms: impaired sensory and motor functions of the extremities, cognitive deterioration, visual dysfunctions, paresthesia, and fatigue. Within 10–15 years of diagnosis, 80% of MS patients face gait problems because of these disorders, which often limit their social life or lead to depression [3–6]. To support mobility, patients use mobility assistive technology (MAT), such as wheelchairs, scooters, walkers, canes etc.

In addition to personal obstacles, MS imposes a huge burden on the healthcare system and community economy due to the high costs of health care facility management, medicinal products used, home care and caregivers, and loss of productivity. According to the review of cost-of-illness studies conducted by Paz-Zulueta *et al*. [7], the mean annual total cost per patient was 40,303€ (59,611€ for advanced course of disease) in 2015, while the respective value was estimated to be $ $65,612in the USA in 2019 [8]. In earlier, less severe stages of the disease, drug costs are the main source of costs, while in the later stages of the disease, loss of productivity due to short- or long-term sick leave, disability pension, inpatient and outpatient care costs account for most of the costs [7,9,10].

Although the etiology of MS is not fully elucidated, it is known that genetic and environmental factors, as well as autoimmunity play a role in the pathophysiology of MS [11]. Three types of the disease course are diagnosed based on their clinical phenotype: relapsing-remitting MS (RRMS), secondary-progressive MS (SPMS) and primary-progressive MS (PPMS). The most common disease course is RRMS, which affects approximately 85% of patients with MS. It consists of consecutive periods of relapses (attacks of new or increasing neurologic symptoms) and remission (partial or full recovery). Relapse terms are mainly characterized by white matter lesions resulting from inflammation and demyelination of neurons [12,13]. SPMS occurs gradually with the neurological worsening of RRMS within 10–20 years. This type of MS is often diagnosed retrospectively due to the uncertainty of its transition from RRMS [14,15]. Increased axonal loss, cortical demyelination and insufficient remyelination are typical of this advanced stage [12,13]. PPMS makes up for 10–15% of MS patients and it is characterized by a constant exacerbation of neurological disability from the disease onset. Cortical demyelination, axonal loss, microglial activation, and atrophy in the white and grey matter are more common in PPMS [16].

Current therapies for MS include disease modifying therapies (DMTs). These immunosuppressant drugs modulate immune cell activity and reduce the number of relapses, but do not cure the disease. Additionally, certain DMTs have the potential of serious adverse effects (SAEs), such as cerebrovascular diseases, infections, secondary autoimmunity, and an increased risk of malignancies due to general immune system suppression [17]. Antigen-specific therapies could be an optimal solution for avoiding such adverse effects (AEs), as they silence only those pathways that contribute to the disease pathogenesis. Although the target antigen in MS is still unknown, proteins within the myelin sheath, such as: RAS guanyl releasing protein-2 (RASGRP2), glial cell adhesion molecule (GlialCAM), myelin phosphatide protein (MBP), myelin oligodendrocyte glycoprotein (MOG) and proteolipid protein (PLP) seem to be relevant targets of the autoimmune response in MS [18–21]. Recently, 4 new relevant antigens have been revealed: fatty acid–binding protein 7, prokineticin-2, reticulon-3, and synaptosomal-associated protein 91 [22].

In recent years, several strategies to induce antigen-specific tolerance have been evaluated in clinical trials for patients with MS [23], involving engineered T cells (or dendritic cells loaded with specific antigens [19,21]), plasmid DNA vaccines encoding 1 or more myelin antigens [24,25], or myelin antigens (peptide-based therapy) [26–29]. Currently, there are no European Medicine Agency (EMA)- and Food and Drug Administration (FDA)-approved antigen-specific tolerance-inducing treatments for MS, although there are many preclinical animal studies and an increasing number of clinical studies assessing their efficacy and safety. The aim of this systematic review is to evaluate the safety and efficacy of tolerance-inducing therapies compared to placebo or other available comparators in MS patients.

A preliminary search for existing systematic reviews on the topic efficacy of antigen-specific therapeutic approaches in adult patients with MS was completed in PROSPERO, JBI Evidence Synthesis, Epistemonikos, and the Cochrane Database of Systematic Reviews. The initial search was conducted in March 2021, and repeatedly search again on 5th February 2024. We did not find any systematic review published on this important topic.

### Review question(s)

What is the efficacy of antigen-specific therapeutic approaches in adult patients with MS?

Are the antigen-specific tolerance-inducing therapeutic approaches safe in adult patients with MS?

### Inclusion criteria

#### Participants.

The review considered studies that include adults (older than 18 years of age) with MS (either type: RRMS, SPMS, PPMS).

#### Interventions.

This review considered studies that evaluated antigen-specific cell- (T-cell- and tolerogenic dendritic cell-) based interventions, nanoparticle-based interventions, DNA-based interventions, and protein-peptide based interventions.

#### Comparators.

This review considered studies that compared the intervention to placebo or other comparators.

#### Outcomes.

This review considered studies that included the following outcomes:

1 Safety of the antigen-specific therapeutic interventions. This was our primary outcome and was determined by the number and proportion of patients with at least one AE and SAE related to the intervention, and treatment discontinuations due to AEs. Routine laboratory (blood count, liver function test, kidney function test, urine analysis, ECG, vital signs etc.), and physical tests were assessed for safety of intervention.
2 Clinical efficacy of the therapies. This outcome was measured by:

- Established scales, e.g., Expanded Disability Status Scale (EDSS), Multiple Sclerosis Functional Composite (MSFC), Multiple Sclerosis Impact Scale (MSIS29), Scripps Neurological Rating Scale (SNRS), timed 25-Foot Walk (FWT), nine-hold peg test (9HPT), paced auditory serial addition test (PASAT) scores;
- The number of relapses by Annualized Relapse Rate (ARR): ARR was defined as the total number of relapses divided by the total person-time at risk of relapse;
- Magnetic resonance imaging (MRI) results (e.g., by measuring number of new gadolinium-enhancing lesions and number of new/enlarging T1, T2 lesions).

3 The planned secondary outcomes were safety endpoints the number/proportion of withdrawals due to AEs/SAEs, and the proportion of patients with an AE related to the intervention.

#### Types of studies.

Since antigen-specific tolerance inducing approaches for MS treatment form a relatively new field of translational research, this review considered both experimental and quasi-experimental study designs, including randomized controlled trials (RCT), non-randomized controlled trials, before- after studies (also called pre-post studies) and interrupted time-series studies. Observational studies (cross-sectional studies, cohort studies, case studies/reports etc.), review articles, systematic reviews, meta-analysis, preclinical, and pilot studies were excluded. Studies with less than 10 patients were also excluded.

## Methods

Our systematic review was conducted in accordance with JBI methodology for systematic reviews of effectiveness evidence [30]. This systematic review’s title was registered with JBI (date registered April 2021). The protocol of this review was registered on PROSPERO with registration number CRD42021236776. The registration record was not checked for eligibility and was automatically published exactly as submitted, due to the focus of PROSPERO on COVID-19 pandemics. There are no nuances or deviations from protocol within the conduct of systematic review.

### Search strategy

The search strategy aimed to locate both published and unpublished studies. An initial limited search of PubMed, Web of Science and Cochrane was undertaken to identify articles on the topic. The text words contained in the titles and abstracts of relevant articles, and the index terms used to describe the articles were used to develop a full search strategy for PubMed (see S1 Appendix in the Supporting Information). The search strategy, including all identified keywords and index terms, was adapted for each included database and/or information source. The reference list of all included sources of evidence was screened for additional studies. Only studies published in English language were included without any date limitation.

The exact dates of the initial searches are given in S1 Appendix in Supporting Information, but as we did not finish our review in a reasonable time, we performed the search again on 5th February 2024. Results for these searches are given in S2 Appendix in the Supporting information.

### Information sources

The databases to be searched included medical literature: MEDLINE via PubMed, Web of Science, The Cochrane Library, CINAHL, Google Scholar, and websites of clinical trial registrations, including ClinicalTrials.gov, WHO International Clinical Trials Registry Platform. To identify additional studies, a manual search for relevant review articles and reference lists of included articles was also performed.

### Study selection

**Fig 1:**
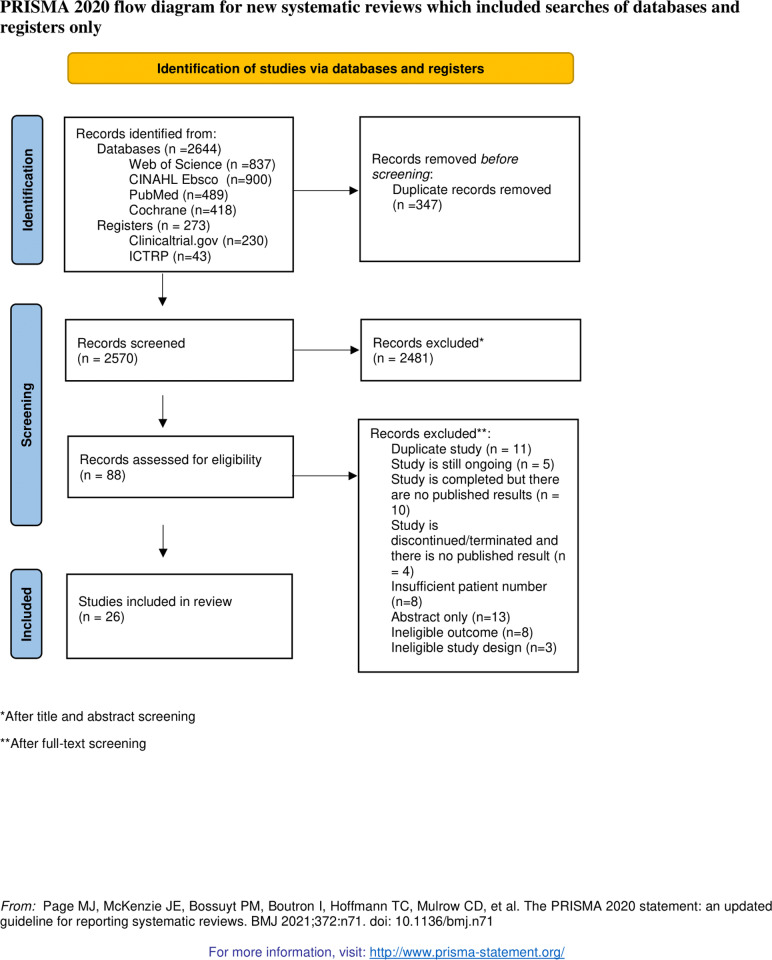
PRISMA flowchart diagram presenting the selection of eligible studies.

Following the search, all identified citations were collated and uploaded into EndNote X9 (Clarivate Analytics, PA, USA) and duplicates were removed. Following a pilot test, titles and abstracts were screened by two independent reviewers (H.K.Ö. and D.M.) for assessment against the inclusion criteria for the review. Potentially relevant studies were retrieved in full, and their citation details imported into the JBI System for the Unified Management, Assessment and Review of Information (JBI SUMARI) (JBI, Adelaide, Australia) [31]. The full text of selected citations was assessed in detail against the inclusion criteria by two independent reviewers (H.K.Ö. and D.M.). Reasons for exclusion of papers at full text that do not meet the inclusion criteria were recorded and reported in the systematic review. Any disagreements that arose between the reviewers at each stage of the selection process were resolved through discussion, or with a third reviewer (O.S.). The results of the search and the study inclusion process was reported in full in the final systematic review and presented in a Preferred Reporting Items for Systematic Reviews and Meta-analyses (PRISMA) flow diagram [32] (Fig 1).

### Assessment of methodological quality

Eligible studies were critically appraised by two independent reviewers (H.K.Ö. and D.M.) at the study level for methodological quality in the review using standardized critical appraisal instruments from the Joanna Briggs Institute for experimental and quasi-experimental studies. Authors of the papers were contacted to request missing or additional data for clarification, where required. Any disagreements that arose were resolved through discussion, or with a third reviewer (O.S.). Given the limited quantity of expected literature in this field, studies were not excluded based on low methodological quality and high risk of bias; rather, study quality was considered when analyzing and interpreting results.

### Data extraction and synthesis

Data were extracted from studies included in the review by two independent reviewers (D.M. and H.K.Ö.) using the standardized data extraction tool. The data extracted included specific details about the populations (e.g., number, age, group and type of MS) study methods (e.g., RCT, non-RCT), interventions (e.g., DNA-based, cell-based, protein/peptide- based), how the study was sponsored, and outcomes (reported clinical outcome measures of efficacy and safety such as AEs, SAEs, MRI results) of significance to the review objective. Any disagreements that arose between the reviewers were resolved through discussion, or with a third reviewer (O.S.). Authors of papers were contacted to request missing or additional data, where required.

The efficacy outcomes (ARR, MRI data and EDSS scores) were presented in a highly inconsistent manner and were often missing. Namely, some studies reported mean values with or without standard deviation, others reported medians; certain studies reported only number of patients showing clinical improvement, etc. Therefore, it was not possible to perform meta-analysis for the efficacy outcomes using either categorical or continuous data.

Data for safety were categorical data, which were described as numbers or percentages. For AEs in all studies, including both RCTs and non-RCTs, overall proportions and 95% confidence intervals were calculated by meta-analysis for proportions. This was not possible for SAEs, because most of the studies did not report any SAEs. RCT were pooled in comparative meta-analysis using the Mantel-Haenszel method. In cases of 0 events in one arm of trial, a fixed value of 0.5 was added to all cells of study results. Effect sizes were expressed as risks ratios (RR) and their 95% confidence intervals. Heterogeneity was assessed statistically using the standard chi squared and I squared tests. Statistical analyses were performed using fixed- and random-effects models for smaller (less than 5) and higher number of studies (more than 5), respectively [33]. Where statistical pooling was not possible, the findings were presented in narrative form including tables and figures to aid in data presentation where appropriate. For visualization of the results, forest plots were used.

Meta-analyses were performed separately for therapies based on DNA, protein/peptide and cells. In certain cases, there were large differences in study sample sizes within these groups, so additional subgroup analyses were performed based on sample size and quality of studies (critical appraisal score>50%). Consequently, sensitivity analyses were conducted to assess whether sample size or quality of studies affected the results of the meta-analyses.

To minimize the risk of bias due to missing results in a synthesis, particularly from reporting biases in meta-analysis, we have looked for selective reporting: we checked the primary studies for discrepancies between protocols (where possible, from ClinicalTrials.gov) and reported outcomes in the publications. Missing outcomes that were pre-specified or inconsistently reported outcomes across studies may signal selective reporting.

### Assessing certainty in the findings

The Grading of Recommendations, Assessment, Development and Evaluation (GRADE) approach for grading the certainty of evidence was followed and a Summary of Findings (SoF) was created using GRADEPro-GDT [34]. The SoF presented the following information where appropriate: absolute risks for the treatment and control, estimates of relative risk, and a ranking of the quality of the evidence based on the risk of bias, directness, heterogeneity, precision and risk of publication bias of the review results. The safety outcomes reported in the SoF were frequencies of the AEs and SAEs in DNA-, protein- and T cells-based therapies.

## Results

### Studies inclusion

The results of the search are shown in the Prism diagram (Fig 1) and in S1 Table in the Supporting Information. The search yielded 2644 results and in total 26 studies were included in the final analysis. Twelve studies were RCTs and 14 were quasi-experimental. For studies which were still ongoing or were finished, but the results were still not published, we have contacted the researchers and asked for an update on the studies. For a study by Zubizarreta, et al. 2019. [21] ewe have received additional data for 5 patients (in total 13 patients). Moreover, for a still ongoing phase 1/2 study to evaluate the safety and efficacy of ATA188 in subjects with progressive multiple sclerosis (ClinicalTrials.gov Identifier: NCT03283826), we have received preliminary data from the authors and these data were also included in the analysis. Finally, publication by Chataway et al., 2018 [29]. Included 2 quasi-randomized studies, so we have analyzed these independently (designated as Chataway 2018a and Chataway 2018b).

The updated search did not find any relevant result, with the exception of a conference paper which presented updated findings from the study mentioned above (Phase 1/2 study to evaluate the safety and efficacy of ATA188 in subjects with progressive multiple sclerosis (ClinicalTrials.gov Identifier: NCT03283826).

### Discontinued studies

There was only one study that was discontinued early, due to high frequency of allergic reactions [35]. However, a considerable number of studies were still officially ongoing, despite the estimated completion date having passed, or had been completed, yet their results had not been posted (S1 Table in the Supporting Information). We reached out to all the responsible researchers regarding the studies’ outcome, but unfortunately, we did not receive any response.

### Studies and subjects’ characteristics

All reports included data mainly from phase I or phase II clinical trials, including 6 dose-escalating studies. There was only one phase III clinical trial [36]. In total, there were 1427 subjects from the RCTs, and 314 from non-RCTs. Sample size of studies ranged from 10 to 612 patients. From RCTs, most of the studies involved subjects suffering from RRMS and SPMS [37–39], RRMS or clinically isolated syndrome (CIS) [40], RRMS only [35,41–43], progressive MS [44], RPMS only [45], SPMS only [36,46]; from non-RCTs, studies included subjects with RRMS [29,47,48], RRMS or SPMS [49–51], progressive MS [52–54] or any type of MS [21,55,56].

Detailed characteristics of the subjects included in the final analysis are presented in Table 1.

**Table 1:** Characteristics of included studies - randomized controlled trial form.

Study	Country		Setting/context		Participant characteristics		Groups	Duration/Dose of Treatment	Outcomes measured	Description of main results		Date of extraction		Extractors	
**DNA-based treatments**															
Bar-Or, et al. 2007.	Canada, USA		Phase I/II randomized, double blind, placebo-controlled trial		Subjects with RRMS or SPMS; aged 18 + years; EDSS score 2.5–6.5; had either 1–5 Gd+ lesions on MRI, a clinical relapse within 2 years prior to screening, or disease worsening in the previous 2 years.		Intervention 1: i.m. DNA vaccine encoding MBP (BHT-3009) (n = 9) with 3 cohorts (0.5 - 1.5- 3 mg, n = 9) Intervention 2: BHT-3009 + atorvastatin calcium (n = 9) with 3 cohorts (0.5 - 1.5 - 3 mg) vs. placebo (saline injections or placebo tablets, n = 12). 10 of these placebo patients reallocated to BHT-3009 with or without 80 mg atorvastatin p.o. after the RCT part.	4 injections at weeks 1, 3, 5, and 9	Efficacy: MRI (No. and V of Gd+ lesions), ARR Safety: AEs, neurological assessments (RR, EDSS), MRI (Gd + and T2 lesions), and standard lab. evaluation of hematological, renal, and liver function; analysis of antigen-specific immune responses	Efficacy: no statistically significant differences between the groups in ARR, positive trends regarding MRI results were seen in the treatment groups Safety: no differences between the groups for AEs and SAEs.		February 2022		D.M. and H.K. Ö.	
Garren, et al. 2008.	USA, Czech Republic, Serbia, Poland, Switzerland		Phase II randomized placebo-controlled study		Subjects with RRMS aged 18–55 years; EDSS scores 0–3.5; had one or more relapses within the previous year, had less than five Gd+ lesions on screening MRI scan		Intervention: DNA vaccine encoding MBP (BHT-3009) - 0.5 mg (n = 96) and BHT-3009–1 mg (n = 84) vs. placebo (n = 87)	i.m. injections given at weeks 0, 2, 4, and every 4 weeks thereafter until week 44.	Efficacy: EDSS, MSFC, MRI (occurrence of new Gd+ lesions, total No. and V of new Gd+ lesions, T2 lesion V change, mean 4-week rate of new T2 lesions throughout the study), time to first relapse, and the proportion of patients with worsening MSFC scores Safety: AEs, routine clinical laboratory testing of blood chemistry, hematology, urine analysis.	Efficacy: in group receiving 0.5 mg MBP reduction of No. and V of new Gd+ lesions was observed, but not in the groups receiving 1.5 mg MBP. No differences in EDSS and MSFC. Safety: no difference between the groups for AEs and SAEs.		February 2022		D.M. and H.K. Ö.	
**Peptide-based treatments**															
Bourdette, et al. 2005.	USA		Phase I/II randomized, partially blinded study		Subjects with RRMS or SPMS aged 18–60, with EDSS scores 0–6.5; at least one clinical relapse or a brain MRI scan with at least one Gd+ lesion within the preceding 24 months		Intervention: i.m. 100 µg TCR peptides ((BV5S2, BV6S5 and BV13S1)/IFA (n = 16) vs. TCR peptides/saline (n = 15) vs. placebo IFA (n = 6)	TCR peptides: injections received at weeks 1, 2, 3, 4, 8, 16, and 20; TCR peptides/IFA: at weeks 1, 4, 8, 12, 16 and 20	Efficacy: EDSS, T25-FW, and 9HPT, brain MRI (% of subjects with active scans and average No. of Gd+ lesions) Safety: AEs, SAEs	Efficacy: TCR peptide responder group tended to have less disease activity (observed by MRI) by week 24, although the differences did not achieve statistical significance; no differences in EDSS, T25-FW and 9HPT between the groups. Safety: no difference between the groups for AEs and SAEs.		February 2022		D.M. and H.K. Ö.	
Freedman et al. 2011.	Canada		Phase III randomized double-blind, placebo-controlled study		Subjects with SPMS aged 18–65 years; EDSS score 3.5–6.5, and a Kurtzke pyramidal or cerebellar system subscore 3.		Intervention: MBP8298 500 mg - two cohorts DR2 + /DR4+ (n = 261) and DR2-/DR4- (n = 41) vs. placebo (sterile water/saline) - two cohorts: DR2 + /DR4+ (n = 218) and DR2-/DR4- (n = 43)	4 i.v. injections at months 0, 6, 12 and 18	Efficacy: EDSS, MSFC, MRI (new T2 or enlarging T2 lesions, T1 Gd + , lesion burden (T2 burden of disease), and brain V), QOL. Safety: AEs, SAEs, laboratory tests, vital signs, ECG, and physical examinations.	Efficacy: no significant differences between groups in any clinical endpoint. Safety: no difference between the groups for AEs and SAEs.		February 2022		D.M. and H.K. Ö.	
Goodkin, et al. 2000.	USA		Phase I, placebo-controlled, double-masked, dose- escalation study		Subjects with SPMS aged 21–60 years; EDSS score 37.5		Intervention: solubilized complex comprised of human leukocyte antigen—DR2 with MBP_84–102_ (AG284) in escalating doses: 0.6, 2.0, 6.0, 20.0, 60.0, 105.0, and 150.0 mg/kg body weight, n = 25 vs. placebo (0.05% n-dodecyl-b-D-maltoside, n = 8)	3 i.v. infusions given every second day	Efficacy: the EDSS, 9HPT, MRI: Gd+ lesions Safety: AEs	Efficacy: no significant treatment effect was detected for any of the clinical measurements. Safety: The frequency of AEs was similar in both groups.		February 2022		D.M. and H.K. Ö.	
Kappos, et al. 2000.	Switzerland, Italy, USA, Canada,		Phase II randomized double-bcontrolled study		Subjects with RRMS aged 18–55 years; EDSS score 0–6; at least 1 or more documented relapses in the year before the study onset		Intervention: altered peptide ligands (APL -3, derived MBP_(83–99)_) cohorts (5 mg, n = 36; 20 mg, n = 36; 50 mg, n = 35) vs. placebo (physiological buffer, n = 35)	Weekly s.c. injections received for 4 months	Efficacy: MRI (No. of new Gd+ lesions and V of the Gd+ lesions), ARR, EDSS. Safety: AEs	Efficacy: no difference in the frequency of relapses and new Gd+ lesions in any of the groups; in patients completing the study the V and No. of Gd+ lesions were reduced at a dose of 5 mg. Safety: hypersensitivity reactions were found in 9% of the patients (**trial suspended**).		March 2022		D.M. and H.K. Ö.	
van Noort, et al. 2015.	Netherlands, Bulgaria		Phase IIa randomized, placebo-controlled, double-blind, exploratory, dose-ranging study		Subjects with RRMS aged 18–55 years; 1 or more Gd + T1 MRI lesions; having had at least one clinical relapse over the previous year or two relapses over the past two years, at least one Gd+ lesion, EDSS score ≤5.5		Intervention: 3 cohorts of alpha B-crystallin (Hsp95): 7.5 mg (n = 8); 12.5 mg (n = 8); 17.5 mg (n = 8) vs. placebo (PBS, n = 8)	3 i.v. injections given at months 0, 2, 4; patients were followed up to week 48	Efficacy: Gd + T1-weighted and T2-weighted brain MRI scans, EDSS and MSIS-29 scores, ARR Safety: AEs, vital signs, ECG, hematology, biochemistry, and urinalysis parameters.	Efficacy: clinical endpoints did not differ significantly between the groups. Lower doses of HspB5 led to a 76% reduction in both No. and total V of active MRI lesions at 9 months. Safety: no difference between the groups for AEs and SAEs.		March 2022		D.M. and H.K. Ö.	
Yadav, et al. 2012.	USA		Phase I double-blind, placebo-controlled, dose-escalation study		Subjects with RRMS or SPMS; aged 18–65; EDSS scores 0–6.5 HLA-DR2 positive		Intervention: Consecutive cohorts with doses of RTL1000: 2 mg, n = 4; 6 mg, n = 7; 20 mg, n = 4; 60 mg, n = 7; 100 mg, n = 3; 200 mg, n = 1 vs. placebo (tris buffer solution, n = 11)	Single i.v. infusion	Safety: AEs, ECG, vital signs, blood chemistries, CBC, and antibodies to RTL1000, MOG- 35–55 peptide, and HLA-DR2, EDSS, 25-foot timed walk, 9HPT, MRI.	Safety: all subjects tolerated the 2–60 mg doses of RTL1000. Doses ≥100 mg caused hypotension and diarrhea in 3 of 4 subjects, leading to discontinuation of further enrolment of patients for these doses.		March 2022		D.M. and H.K. Ö.	
Walczak et al. 2013.	Poland		Double-blind, placebo-controlled study		Subjects with RRMS aged 18–55 years; EDSS score 0–5.5, and 1 or more relapses within the previous year.		Intervention: myelin peptide skin patch- mixture of 3 myelin peptides (MBP85–99, MOG35–55, and PLP139–155): 2 cohorts (1 mg, n = 16) and 10 mg, n = 4)) vs. olacebo (PBS, n = 10)	t.d. patch carried during a year (changed once per week for 4 weeks and then once per month for 11 months)	Efficacy: cum. No of active Gd+ lesions, mean V of Gd+ lesions; a cum. No. of new T2 lesions; and T2 lesion and T1 lesion V change from baseline; ARR, the proportion of relapse-free patients, and the proportion of patients with 3 months of confirmed disability worsening on the EDSS at the end of the study. Safety: AEs, SAEs	Efficacy: compared with placebo, treatment with a myelin peptide skin patch (1 mg) showed a 66.5% reduction in the cum. No of Gd+ lesions and lower ARR. Safety: No differences in the distribution of AE (exception is local reaction in the area of the skin patch). No SAEs were reported.		March 2022		D.M. and H.K. Ö.	
Warren, et al. 2006.	Canada		Phase II double-blind, placebo- controlled clinical study		Subjects with PMS; aged 32–60 years; EDSS scores 3–7.5, MRI of the brain and spinal cord with lesions characteristic of MS, one or more relapses in the previous 2 years		Intervention: 500 mg MBP8298 (n = 16) vs. placebo/saline (n = 16);	4 i.v. injections at months 0, 6, 12, 18 Plus, in the follow-up, the patients who wanted, continued to receive th. every 4 months during 2 years and then every 6 month till the month 84.	Efficacy: EDSS, 22-m timed walk test, MRI (unique new, unique enlarging, new proton density, enlarging proton density, new enhancing, and spinal cord lesions) Safety: routine clinical laboratory testing (CBC, heart panels, liver enzymes, kidney panels, CSF analyses), AEs	Efficacy: no difference between the groups for EDSS, MRI, and 22 m-timed walk test; a significant delay of EDSS progression in a major subgroup of treatment group with HLA haplotypes DR2 and/or DR4. Safety: no difference between the groups for AEs and SAEs.		March 2022		D.M. and H.K. Ö.	
**T cells-based treatments**															
Karussis, et al. 2012.		Israel		Phase II double-blind, sham-controlled study	Subjects aged 18–60 with RPMS and EDSS: 3–7; severe relapses during the year prior to inclusion, MRI of the brain with at least 5 lesions in the white matter (T2-weighted imaging)		Intervention: T cell vaccination (specific for 9 antigens which are sequences from MBP, MOG or PLP) 10–30 million attenuated T-cells, n = 19) vs. placebo (saline, n = 7)	4 s.c. injections on days 1, 60, 90 and 180	Efficacy: EDSS, the timed 10-meters walking, the 9-hole peg test for hands dexterity, PASAT), ARR, MRI: total burden of hyperintense lesions in T1- and T2-weighted imaging, the degree of cortical atrophy and axonal loss Safety: SAEs, AEs, vital signs, ECG, CBC		Efficacy: significant decrease in EDSS scores and in 10-meter walking time were observed in the TCV group. Significantly more patients in the TCV group remained relapse-free compared to the placebo group. MRI parameters did not change significantly. Safety: no difference between the groups for AEs and SAEs.		March 2022		D.M. and H.K. Ö.
Fox et al. 2012.		USA		Phase IIb randomized placebo-controlled study		Subjects with RRMS or CIS aged 18–55 years; EDSS score 0–5; one Gd+ lesion or 2 lesions consistent with MS	Intervention: Tovaxin® (2x 10^5^ *in vitro* expanded myelin-reactive T-cells, against MBP, MOG or PLP, n = 100) vs. placebo (sterile saline + human serum albumin, n = 52)	5 s.c. injections at weeks 0, 4, 8, 12, and 24	Efficacy: MRI: cum. No of Gd + T1 lesions and new Gd+ lesions, change in T2-weighted lesion V, ARR and EDSS; Safety: CBC, serum blood chemistry, urinalysis, vital signs, physical and neurological exams		Efficacy: no differences groups regarding clinical or radiographic endpoints. In subjects with ARR > 1 at baseline, there was higher reduction in relapses and EDSS in Tovaxin® group compared with placebo. Safety: no difference between the groups for AEs and SAEs.		March 2022		D.M. and H.K. Ö.

Gad+ = gadolinium-enhancing; CBC = complete blood count; CIS = clinically isolated syndrome; cum. No = cumulative number; ECG = electrocardiogram; IFA = Incomplete Freund’s adjuvant; i.m. = intra-muscular; MBP = myelin phosphatide protein; MOG = myelin oligodendrocyte glycoprotein; MRI = magnetic resonance imaging; MSFC = MS Functional Composite Score; MSIS29 = multiple sclerosis impact scale 29; PLP = proteolipid protein; PBS = phosphate buffer saline; PASAT = Paced auditory serial addition test; p.o. = per os; QOL = quality of life; RRMS = relapsing-remitting multiple sclerosis; RR = relapse rate; SPMS = secondary progressive multiple sclerosis; TCR = T cell receptor; t.d. = transdermal; th. = therapy; T25-FW = 25-foot timed walk, V = volume; 9HPT = Nine Hole Peg Test.

The majority of the studies were funded by a profit manufacturer (N = 15), some reported an academic sponsor/research center (N = 7), and others did not report the sponsor explicitly (N = 6). Studies were conducted in Europe, United States of America, Canada, Israel, and Australia.

### Therapies

In 12 RCTs, therapy consisted of products based on T cells (2 trials), DNA-based technology (2 trials), and peptide-based products (8 trials). Both trials regarding DNA – based technology studied BHT 3009, which is a MBP-coding gene [37,41]; both T cell-based vaccines evaluated attenuated T cells specific for myelin peptides, such as MBP, MOG or PLP [40,45]. Targeted peptides in the trials were TCR peptides [38], TCR ligand [39], MBP [35,36,44], complex of DR2 and MBP [46], mixture of MBP, PLP and MOG [43], and alpha B-crystallin [42]. In all trials, placebo was the control (in the study by Bourdette et al. 2005 [38], there were 3 groups: placebo, peptides dissolved in incomplete Freund’s adjuvant (IFA) and the same peptides administered without IFA). Administration route included intramuscular (i.m.) [37,38,41], intravenous (i.v.) [36,39,42,44,46], subcutaneous (s.c.) [35,40,45], and transdermal (t.d.) application [43]. The length of treatment varied highly from one day (single i.v. infusion) to 84 months.

Regarding non-RCTs, there were 8 T cell-based studies and 6 studies evaluating peptide-based therapies. Treatments were administered s.c. [29,47,49–51,57], i.m. [55,56], p.o. [58], intradermally (i.d.) [29,52], and i.v [21,48,53,54]. Treatments including T cells targeted Epstein-Barr virus (EBV) [53,54], whole myelin [57], myelin peptides MOG, PLP, MBP [21,50], or MBP and MOG [47], MBP only [51] or included T regulatory (Treg) cells [48]. From peptides, regions of MBP [29,59], peptides from V region of TCR [52,55,56], and bovine myelin [58] were assessed. Length of treatment varied from 4 weeks up to 5 years.

### Critical appraisal results – quality assessment

**Fig 2:**
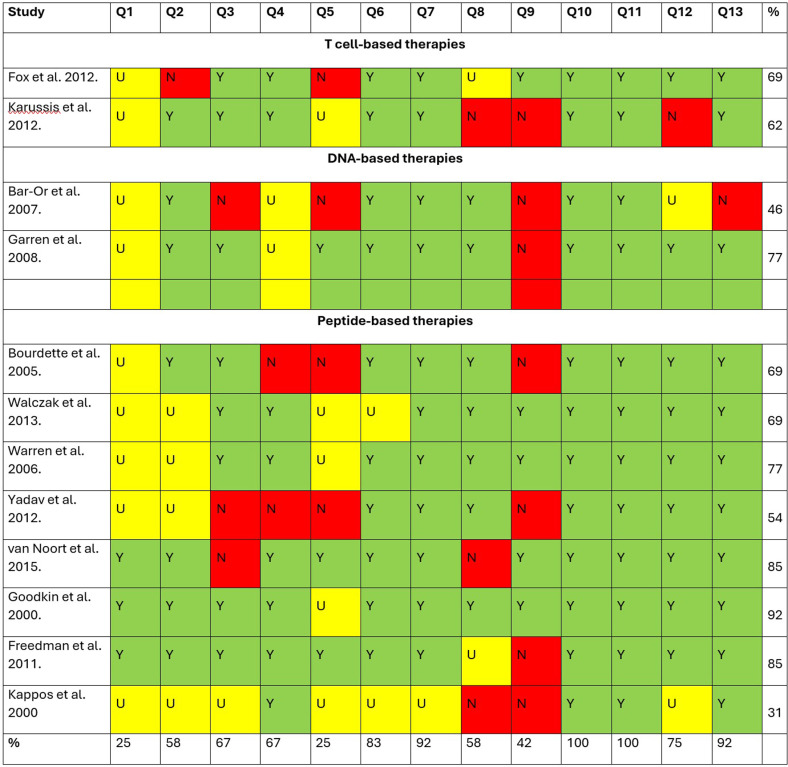
Critical appraisal of randomized controlled trials. Y = yes; N = no; U = uncertain. JBI critical appraisal checklist for randomized controlled trials: Q1: Was true randomization used for assignment of participants to treatment groups? Q2: Was allocation to treatment groups concealed? Q3: Were treatment groups similar at baseline? Q4: Were participants blind to treatment assignment? Q5: Were those delivering treatment blind to treatment assignment? Q6: Were outcome assessors blind to treatment assignment? Q7: Were treatment groups treated identically other than the intervention of interest? Q8: Was follow-up complete, and if not, were strategies to address incomplete follow-up utilized? Q9: Were participants analyzed in the groups to which they were randomized? Q10: Were outcomes measured in the same way for treatment groups? Q11: Were outcomes measured in a reliable way? Q12: Was appropriate statistical analysis used? Q13: Was the trial design appropriate, and any deviations from the standard RCT design (individual randomization, parallel groups) accounted for in the conduct and analysis of the trial.

**Fig 3:**
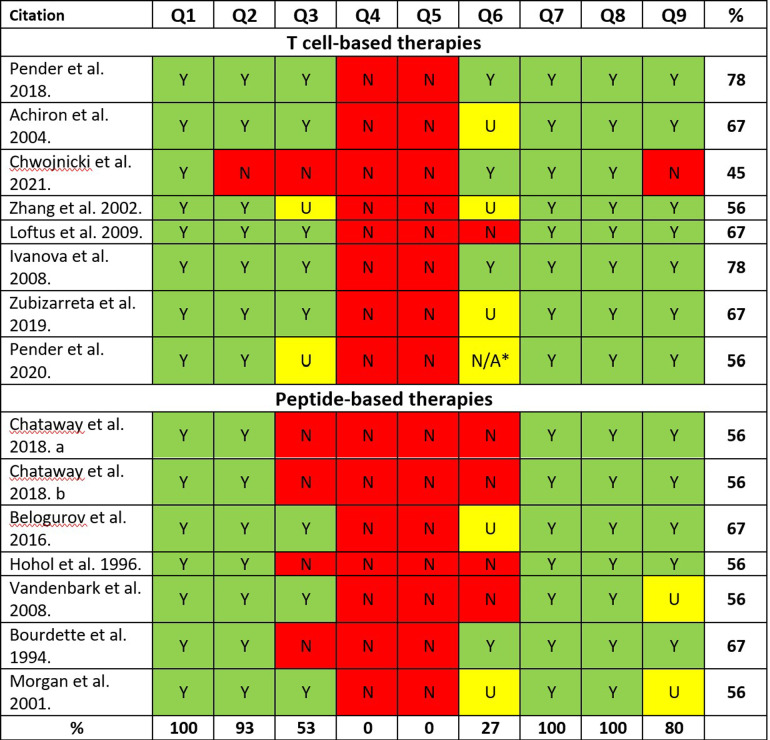
Critical appraisal of quasi-experimental studies. Y = yes; N = no; U = uncertain; Y = yes; N = no; U = unclear. JBI critical appraisal checklist for quasi-experimental studies (non-randomized experimental studies): Q1: Is it clear in the study what is the “cause” and what is the “effect” (i.e., there is no confusion about which variable comes first)? Q2: Were the participants included in any comparisons similar? Q3: Were the participants included in any comparisons receiving similar treatment/care, other than the exposure or intervention of interest? Q4: Was there a control group? Q5: Were there multiple measurements of the outcome both pre and post the intervention/exposure? Q6: Was follow up complete and if not, were differences between groups in terms of their follow up adequately described and analyzed? Q7: Were the outcomes of participants included in any comparisons measured in the same way? Q8: Were outcomes measured in a reliable way? Q9: Was appropriate statistical analysis used? *Ongoing study.

In total, 26 studies were critically appraised for methodology quality using either the quasi-experimental studies or the RCT critical appraisal checklist, appropriately to the study design (please see Figs 2 and 3 for full methodological quality assessment). Critical appraisal scores for the RCTs were in the range 31% to 92% (Fig 2). Most important methodological concerns were associated with true randomization and treatment-blinding for participants, as only 25% of the critically appraised studies had satisfactory appraisal for these requirements. Therefore, most of the studies were at a moderate/severe risk of bias.

For quasi-experimental studies, critical appraisal scores were in the range 45% to 78% (Fig 3). All studies included pre-post design. The main source of bias were the lack of control group and multiple measurements of the outcome both pre and post the intervention/exposure in all studies. None of the studies was positively appraised for these requirements. Moreover, in 73% of the studies follow up was incomplete, mostly because the authors failed to report the reasons for loss to follow up. Another confounding factor, which was present in approximately half of the studies, was the simultaneous use of immunosuppressive therapies in participants.

### Efficacy outcomes

Efficacy outcomes are presented in Tables 1 and 2. Most of the studies were phase I or II, so examination of the treatment efficacy was not their primary goal. Additionally, as previously mentioned, the results of these outcomes were highly inconsistently reported or missing. In both RCTs and quasi-experimental trials, efficacy of the treatments was measured by established scales, ARR and MRI data. Most frequently used scale was EDSS, often accompanied with other scales, such as MSFC, MSIS29, 9HPT, T25-FW, and PASAT. Moreover, very different endpoints of MRI were followed and reported in these studies: some studies looked for new Gd+  T1-weighted and T2-weighted brain MRI scans, others looked for their volumes, certain authors reported their cumulative values before and after the treatment, while others looked at the fraction of patients with active scans before and after the treatment. Therefore, the efficacy of all antigen-specific treatments could not be systematically analyzed and remains ambiguous. In the following section, we narratively present the most important results from the analyzed studies.

**Table 2:** Characteristics of included quasi-experimental studies.

Study	Country	Setting/context	Participant characteristics	Groups	Duration/Dose of Treatment	Outcomes measured	Description of main results	Date of extraction	Extractors of the data
				**Peptide-based treatments**					
Belogurov et al. 2016.	Russia	Phase I, multicenter, open-label, dose-escalating safety, and proof-of-concept study	Subjects with RRMS or SPMS, aged 18–55 years, EDSS 3–5.5 and had > 1 relapse during the previous year, underwent unsuccessful treatment by immuno-modulators	Intervention: 2 cohorts: Xemis®: oligopeptides MBP46–62, MBP 124–139, and MBP 147–170 co-encapsulated in CD206-targeted small mono-lamellar liposomes MBP peptides at doses ascending from 50 μg to 900 μg (n = 20)	6 weekly s.c. injections	Efficacy: EDSS, RR, MRI (No of Gd + T1 lesions and the cum. No of lesions in T2 and FLAIR sequences) Safety: laboratory tests, O_2_ saturation, HR, BP, RsR, temperature, AE, SAE	Efficacy: 85% patients were relapse-free, and EDSS worsened in 21% and improved in 10% of patients; increased No. of active Gad+ lesions at weeks 7 and 10, but not at week 18 Safety: no SAEs, most common AE was local reaction at the site of injection, no changes in laboratory tests and other vital parameters	November 2021	D.M. and H.K. Ö.
Bourdette, et al. 1994.	USA	Open-label single-arm study	Subjects with PMS, aged 37–66 years, having 3.6–7.5 EDSS scores	Intervention: peptides from V region of TCR (Vβ5.2 and Vβ6.1), n = 11	4 weekly i.d. injections of 100 μg followed by doses of 200, 300, 600, 1500 and 300 μg administered every 4 weeks	Safety: clinical (EDSS, AI); CBC, urine analysis, 24 channel chemistry panel; proliferative response to various mitogens and recall antigens	Safety: no proliferative response to various mitogens and recall antigens; no changes in biochemistry analyses; AE: injections site reactions; clinical response: among 7 responders, 1 was improved, 4 were stable and 2 were worse	November 2021	D.M. and H.K. Ö.
Chataway, et al. 2018. a	United Kingdom, Russia	Phase Ib, multicenter, 2-arm, ascending-dose safety and proof-of-principle study	Subjects with RRMS aged 18–55 years, having ≥1 documented relapse in 12 months or 2 relapses in 24 months prior to the study onset), EDSS ≤5.5, positive HLA-DRB1*15	Intervention: ATX-MS-1467 (cocktail of epitopes from 4 regions of MBP), (i.d., n = 20 and s.c. application, n = 22)	8 week-titration period: 25, 50, 100, 400, and 800 μg at 14 ± 3 day intervals followed by 8-week, full-dose period of 800 μg at 14 ± 3 day intervals in 2 cohorts (i.d. and s.c.)	Efficacy: EDSS, MSFC, MRI (new T1 Gad+ lesions, new or enlarged T2 hyperintense lesions, and T1 hypointense lesions) Safety: SAEs, AEs, laboratory values, anti-peptide antibody tests, vital signs, physical examination, neurologic ex-amination, and MRI	Efficacy: a significant 73% decrease in the No. of new or persisting Gad+ lesions in i.d. cohort, slight increase in new lesions in s.c. cohort; decreased V of Gad+ lesions only in i.d. cohort, increase lesion-free patients only in the i.d. cohort Safety: 77% of patients experienced AE which were mild or moderate, number was higher in the i.d. cohort (injection-site reactions), no SAEs	November 2021	D.M. and H.K. Ö.
Chataway, et al. 2018. b	Russia, Latvia	Phase IIa, multicenter, single-arm, proof-of-concept trial	Subjects with RMS aged 18–55 years, having ≥1 documented relapse in the previous 12 months or 2 relapses within the previous 24 months), EDSS ≤5.5, positive HLA-DRB1*15	Intervention: ATX-MS-1467 (cocktail of epitopes from 4 regions of MBP), n = 37	4-week titration period, i.d. injections, the dose from 50 μg on day 1–200 μg on day 15 and 800 μg on day 29; Subsequently, biweekly 800 μg during 16 weeks	Efficacy: No. and V of new T1 and T2 Gad+ lesions; EDSS, MSFC, PASAT scores; ARR Safety: AEs, injection-site reactions, vital signs, clinical laboratory variables, ECGs, and the frequency and timing of premature study termination.	Efficacy: significant decrease in the No. of T1 Gad+ lesions on treatment vs. baseline; no change in EDSS and MSFC scores; PASAT scores improved Safety: 79% of subjects experienced AEs which were mild or moderate	November 2021	D.M. and H.K. Ö.
Hohol, et al. 1996.	USA	Open protocol continuation study of a phase III trial	Subjects with RRMS or PMS, aged 18–54	Intervention: 300 mg of bovine myelin given p.o. (n = 16)	Daily for 3 years	Efficacy: EDSS Safety: AEs, clinical and biochemical laboratory tests	Efficacy: in 13 patients EDSS decreased, in 3 worsened Safety: no AEs, no changes in laboratory measurements	November 2021	D.M. and H.K. Ö.
Morgan, et al. 2001.	USA	Multicenter, open-label I phase trial	Subjects with RRMS and CPMS; aged 28–64 years; EDSS: 1–9; MS diagnosis clinically or laboratory confirmed for more than 1 year	Intervention: BV6S2/6S5 peptide emulsified in IFA- 300 µg (n = 10)	5 i.m. injections administered at 0, 4, 12, 24 and 36 weeks	Efficacy: EDSS Safety: AEs, SAEs, clinical laboratory tests and physical and neurologic examinations.	Efficacy: EDSS scores remained stable Safety: no SAEs, 2 AEs (injection site reactions), no significant changes in any laboratory parameters	November 2021	D.M. and H.K. Ö.
Vandenbark, et al. 2008.	USA	Open-label single-arm study	Subjects with RRMS, PPMS or SPMS, aged 18–75 years, EDSS score <7; definite MS by Modified Poser criteria	Intervention: trivalent TCR peptide (BV5S2, BV6S5 and BV13S1) emulsified in IFA 100 in dose of 100 µg/ml of each peptide (n = 23)	12 i.m. vaccinations administered every 4 weeks	Efficacy: EDSS, FWT, 9HPT Safety: AEs	Efficacy: EDSS: 19 remained stable and 4 worsened Safety: not reported (although mentioned in the Methods section)	November 2021	D.M. and H.K. Ö.
				**Treatments based on T cells**					
Achiron, et al. 2004.	Israel	Open-label, single-arm study	Subjects with RRMS aged 18–60 years; EDSS score ≤6; brain MRI compatible with MS; increase in RR and/or progression of at least 0.5 point of the EDSS score in the year before the study when the patient was under immunomodulatory treatment	Intervention: Autologous MBP- and/or MOG-reactive T cells (up to 1.5 x 10^7^ cells per peptide), n = 20	3 s.c. injections in period of 6–8 weeks	Efficacy: RR, EDSS, MRI Safety: AEs, vital signs, CBC, liver function tests, electrolytes, kidney function tests, and serology for hepatitis A, B, C, and HIV	Efficacy: reduction of RR; significant decrease in the No. and V of active lesions, and T2 lesion burden. Safety: no SAEs, redness at the injection site in 55% of patients	December 2021	D.M. and H.K. Ö.
Bar-Or et al, 2021. (ongoing)	USA, Canada, Australia	Phase I study (open-label, single-arm, sequential dose-escalation study) + Phase II (double-blind, randomized, placebo-controlled dose-expansion period followed by OLE period)	Subjects with PMS, aged 18–60 years, EDSS score 3–7; positive EBV serology, active clinical relapse between given consent and first dose administration	Intervention: ATA188, EBV-targeted T-cells therapy (of 5 × 10^6^, 1 × 10^7^, 2 × 10^7^, and 4 × 10^7^ T cells), n = 24	2 cycles of i.v. injections of ATA188 and followed for 12 months; this is followed by a 4-year open-label extension where subjects are treated annually with one cycle of ATA188	Efficacy (part 1): EDSS, T25FW, 9HPT, FSS, MSIS; MSWS, and MRI (whole brain V) Safety (part 1): AEs, SAEs and clinically significant changes in laboratory tests, ECGs, and vital signs	Efficacy (part 1): 9 subjects reached SDI, 13 participants displayed stable EDSS scores and 4 subjects perceived disability progression. 4 years into OLE, 5 patients preserved clinical improvements with a median duration of improvement of 27.5 months. Additionally, 8 subjects with stable EDSS scores also remained stable for a median of 41.2 months. Safety (part 1): All doses were well-tolerated, with no dose-limiting toxicities. There were no reports of infusion-related reactions, cytokine release syndrome, or graft versus host disease.	December 2021	D.M. and H.K. Ö.
Chwojnicki, et al. 2021.	Poland	Phase Ib/IIa, open-label, two-arm clinical trial	Subjects with RRMS, EDSS score ≤4; aged 18–55 years; having at least one relapse during the last year or at least two relapses in the past 2 years	Intervention 1: CD4^ + ^CD25^high^CD127^ − ^FoxP3^ +^ Treg cells (i.v.)- 40 × 10^6^ Treg cells/kg (n = 11) Intervention 2: Treg cells (i.t.), dose 1.0 × 10^6^ Treg cells/kg (n = 3)	One injection and follow up for 12 months	Efficacy: EDSS, EQ-5D and MSFC FWT, 9-HPT) PASAT, RR, MRI (3D T1-weighted, 3D T2-FLAIR, 3D T2-weighted, Gad + T1) Safety: AEs, CBC, metabolic, kidney, and liver panels, CRP- levels, and urinalysis; MRI	Efficacy: 3/10 i.v. patients deteriorated more than 1 point on the EDSS; no patients in the IT group experienced a relapse. No significant differences in MSFC in both groups. MRI: significantly lower change in the T2 lesion V in the i.t. group compared to the i.v. group. The increase in No. of new T2 lesions was significant in the i.v. group only. Safety: no SAEs, AEs: relapses and the presence of new or enlarging T2 lesions in i.v. group only	December 2021	D.M. and H.K. Ö.
Ivanova, et al. 2008.	Russia	Open-label, single-arm clinical trial	Subjects with RRMS or PMS, aged 18–54; MS confirmed by MRI	Intervention: s.c. vaccine of autologous myelin-reactive T cells in dose 2–4 x 10^7^ cells (n = 28)	Induction (4 injections administered at one-week intervals); supportive treatment included injections given with 1–2-month intervals for 2 years	Efficacy: EDSS Safety: AEs, clinical and biochemical laboratory tests	Efficacy: in 3 patients EDSS decreased, in 16 did not change, in 9 worsened Safety: no AEs, no changes in laboratory measurements	December 2021	D.M. and H.K. Ö.
Loftus, et al. 2009.	USA	Open-label dose escalation study	Patients with RRMS or SPMS; aged 18–65 years; ≥ 1 relapses in the year before enrollment; brain MRI compatible with MS; EDSS score 2–8	Intervention: T-cell vaccine given s.c. (Tovaxin), 3 cohorts (6–9 × 10^6^ MRTC (n = 6), 30–45 × 10^6^ MRTC (n = 5), and 60–90 × 10^6^ MRTC (n = 5))	4 injections at weeks 0, 4, 12, and 20	Efficacy: EDSS, MSIS-29, RR, MRI (cum. No and V of Gd+ lesions in T1 and T2) Safety: AEs, laboratory assessments: blood chemistry, urinalysis; vital signs, neurologic examinations and changes in the brain lesion profile MRI.	Efficacy: reduction in relapses compared to baseline for the M-ITT and evaluable per-protocol analyses were 63.5%, and 85.0% at week 52; MRI lesions were stable while there was an improvement trend in the EDSS and MSIS- 29 following the second injection. Safety: mild or moderate AE unrelated to the vaccine administration (most frequent: injection-site pain and inflammation)	December 2021	D.M. and H.K. Ö.
Pender, et al. 2018.	Australia	Open-label phase I trial	Subjects with PPMS or SPMS, positive EBV serology; aged 18 + years; EDSS score 5–8; progressive neurological deterioration due to MS for at least 2 years	Intervention: Epstein-Barr virus–specific T cell therapy (of 5 × 10^6^, 1 × 10^7^, 1.5 × 10^7^, and 2 × 10^7^ T cells), n = 10 (5 patients with SPMS and 5 patients with PPMS)	4 i.v. injections administered at 2-week intervals	Efficacy: EDSS scores; cognitive and fatigue assessment; screening for depression; QOL blood testing; MRI (T1 and T2 lesions). Safety: O_2_ saturation, HR, BP, RsR, temperature, AE, SAE	Efficacy: 7 patients showed clinical improvement, 3 patients remained stable, and 1 patient showed deterioration of symptoms; compared with baseline, the No. of lesions at week 15 increased in 3 participants (all showed neurological improvement) and decreased in 1 participant Safety: no SAEs and one AE (altered taste, grade 1)	December 2021	D.M. and H.K. Ö.
Zhang, et al. 2002.	USA	Open label single-arm study	Subjects with RRMS or SPMS, EDSS score 1.5–6.5 (for RRMS) and 4–8 (for SPMS); aged 18 + years; having at least one exacerbation in the 2 years prior to study entry for the RRMS cohort.	Intervention: MBP-reactive T cells – 30 x 10^6^ – 60 x 10^6^ cells per s.c. injection (n = 54)	3 injections at 2-month intervals	Efficacy: EDSS, ARR, and MRI lesion activities. Safety: AEs, vital signs and physical examinations	Efficacy: 40% decrease in RR and a minimal reduction in EDSS in RRMS patients; a small increase of EDSS in SPMS patients; MRI showed a stabilization of the lesion activity Safety: no detected AEs associated with the treatment	February 2022	D.M. and H.K. Ö.
Zubizarreta*, et al. 2019.	Spain	Open-label, single-center, multiple ascending- dose phase 1b clinical trial	Subjects with MS (any subtype) aged 18–65 years; EDSS: 3–8.5; > 1 year of disease duration	Intervention: autologous tolDCs loaded with peptides (MBP1, 2, 3, 4, MOG1, 2, PLP1, 2); the cell dose escalation was 50 × 10^6^, 100 × 10^6^, 150 × 10^6^, 300 × 10^6^ cells (n = 13)	3 i.v. injections given at weeks 0, 2 and 4 weeks	Efficacy: ARR, EDSS, MSFC, MRI (No. of new Gad+ lesions, No. of new/enlarging T2-FLAIR lesions) Safety: AEs, vital signs (temperature, BP, HR), blood chemistry, CBC	Efficacy: EDSS and MRI: no signs of disease reactivation, but stability Safety: no SAEs, no changes in laboratory measurements	February 2022	D.M. and H.K. Ö.

*Added data from authors (8 patients from publication + 5 patients from extension). We received the data from the authors via mail in September 2021.

AE = adverse effects; AI = ambulation index; BP = blood pressure; CBC = complete blood count; CPMS = chronic-progressive multiple sclerosis; CRP = C - reactive protein; cum. No = cumulative number; EBV = Epstein-Barr Virus; FSS = Fatigue severity scale; HLA = human lymphocyte antigen; FLAIR = fluid-attenuated inversion recovery; Gad+ = gadolinium-enhancing; HR = heart rate; IFA = incomplete Freund’s adjuvant; i.d. = intradermal; i.v. = intravenous; i.t. = intrathecal; FWT = Timed 25-Foot Walk; MBP = myelin basic protein; M-ITT = modified intention-to-treat; MOG = myelin oligodendrocyte; MRTC = myelin reactive T-cells; MSFC = multiple sclerosis functional composite score; MSIS-29 = Multiple Sclerosis Impact Scale-29; MSWS = MS walking scale; No = number; OLE = open-label extension; PASAT = Paced Auditory Serial Addition Test; PLP = proteolipid protein; PMS = progressive MS; RsR = respiratory rate; RRMS = relapsing-remitting multiple sclerosis; RR = relapse rate; SAE = serious adverse effects; s.c. = subcutaneous; SDI = sustained disability improvement; SPMS = secondary progressive multiple sclerosis; QOL = quality of life; T25FW = 25-foot walk time; V = volume; tolDCs = tolerogenic dendritic cells; TVR = T-cell receptor; 9HPT = Nine Hole Peg Test.

Two RCTs examined the same DNA-based treatment (BHT-3009, a myelin basic protein-encoding plasmid) [37,41]. Studies reported similar results; they observed some positive trends in the several MRI endpoints, including a reduction of occurrence rate of new Gd+ enhancing lesions. However, there was no improvement in ARR, as well as in EDSS scores.

Regarding peptides, none of the RCTs showed any significant clinical benefit quantifying the disability in MS by scales. However, there was one study which examined the treatment with MBP8298 and found it caused a significant delay of EDSS progression in a major subgroup of patients with HLA haplotypes DR2 and/or DR4 [44]. This finding highlighted the importance of genetic background for the potential treatment success. However, this observation was not confirmed in a larger phase III RCT [36]. Regarding MRI results, some studies reported certain favorable findings only in the treatment group, whereas some studies did not report differences between the placebo and the treatment group [36,46]. In the trial examining TCR peptides (BV5S2, BV6S5 and BV13S1), the immunological responders tended to exert less disease activity (scan was considered to be active if there were one or more enhancing lesions) by week 24, although the differences did not achieve statistical significance. Moreover, the treatment with a small heat shock protein alpha B-crystallin (HspB5) resulted in a progressive decline in MS lesion activity, i.e., in a 76% reduction in the number and total volumes of active MRI lesions at 9 months [42]. Finally, in a small study, a one-year t.d. application of the myelin peptide skin patch showed a 66.5% reduction in the cumulative number of active lesions [43]. This study also reported a decline in the ARR in the treatment group only. Regarding quasi-experimental studies examining peptides-based therapies, it is difficult to draw a conclusion in terms of efficacy, as all studies included one-group pretest-posttest design with a small number of patients and mostly intended to assess safety of the therapies (for more details, see Table 2). From these studies, maybe the most distinguished one was examining ATX-MS-1467 (cocktail of epitopes from 4 regions of MBP) [29]. An i.d. route of administration was more favorable than the s.c. route in this study. It resulted in a significant decrease in the number of T1 Gd+ lesions at the end of the study (20 weeks). There was no change in EDSS and MSFC scores, but PASAT scores were improved.

There were 2 RCTs investigating therapies including specific T cells: one examining vaccine with T cells (TCV) against 9 antigens which were sequences of MBP, MOG or PLP [45], while the other looked into the effects of Tovaxin®, the vaccine including *in vitro* expanded myelin-reactive T-cells against MBP, MOG or PLP [40]. In both studies therapies did not affect MRI endpoints, but some beneficial clinical outcomes were reported. There was a significant decrease in EDSS scores and in 10-meter walking time in the TCV group [45]. Significantly more patients in the TCV group remained relapse-free compared to the placebo group. Treatment with Tovaxin® did not display any clinical benefit compared to placebo in terms of ARR and EDSS. However, a post-hoc analysis was performed using data from patients with more active baseline disease (ARR ≥ 1, ARR > 1), all of whom were naïve to DMT before joining the study. This analysis revealed that these patients experienced greater improvement in disability measured by EDSS. A previous smaller quasi-experimental study with Tovaxin®, where patients with ARR > 1 were included, showed similar results [50]. The quality of quasi-experimental studies examining T-cells-based therapies, was similar to the quality of those investigating peptide-base therapies, or slightly better (except for [48]). It is comparably difficult to draw a conclusion with respect to the efficacy of T-cells-based therapies, as all studies included one-group pretest-posttest design with a small number of patients (for more details, see Table 2). It is, however, worth mentioning the novel approach of adoptive T cell therapy against EBV, which has come up as a potential therapeutic strategy for the treatment of MS. Two studies (one is still ongoing) investigating this kind of therapy were included in this analysis [53,54]. In the first study [53], there was a symptomatic improvement (including reducing fatigue and in some cases decline in EDSS score) in 7 of 10 patients. The number of active lesions on MRI increased in 3 patients, but interestingly, all these patients showed symptomatic improvement. In the ongoing study [54], 9 from 24 subjects have reached sustained disability improvement (SDI) which was based on the improvement in EDSS scores or decrease in the time needed to walk 25 feet. Moreover, 13 participants displayed stable EDSS scores and 4 subjects perceived disability progression. After 4 years of follow-up, 5 patients preserved clinical improvements with a median duration of improvement of 27.5 months. Additionally, 8 subjects with stable EDSS scores also remained stable for a median of 41.2 months. Importantly, these patients also showed evidence of increased levels of a biomarker for myelin density, as indicated by magnetization transfer ratios (MTR) on MRI scans. This was a phase I of the clinical trial NCT03283826 and the second part is still ongoing (II phase RCT).

### Safety outcomes

The monitored safety outcomes and the observed main results of the studies are presented in Tables 1 and 2. Specific AEs and SAEs reported in the studies are presented in detail in Table 3. Description of AEs and SAEs in both RCTs and quasi-experimental studies was highly heterogeneous; some studies reported both related and unrelated AEs and SAEs, whereas others stated only that there were no AEs/SAEs related to the treatment; some studies reported specific AEs and SAEs, while the others reported the organ system related to AE/SAE). Unfortunately, only several studies reported the grade of severity of AEs symptoms (mild, moderate, severe). Therefore, we were unable to group AEs into different grades (mild, moderate, severe).

**Table 3:** Safety data reported in studies.

Study	Most common adverse effects	Less common adverse effects	Serious adverse events *	Number of patients who withdrew from study due to AE/SAE
		**Randomized controlled trials**		
Bar-Or, et al. 2007.	Injection site reactions	GIT problems (diarrhea, gas/abdominal pain and discomfort, upset stomach, sore throat), fatigue, dizziness, sweats, gynecologic issues, palpitations, contusion	0	0
Bar Or et al., 2021. (ongoing study)	-**	Runny nose, infections	3 (muscle spasticity, not treatment related, MS relapse, possibly treatment related, fall, not treatment related)	1
Bourdette, et al. 2005.	Injection site reactions	–	None (4 not treatment-related)	Two subjects due to worsening of MS symptoms (neither of these subjects developed an immune response to the IR903 vaccine)
Fox et al. 2012.	Injection site reactions	–	Diplopia, lower limb fracture, arthralgia, intervertebral disc degeneration, and muscular weakness – listed as SAEs due to the need of treatment, but not severe and not related to the treatment	0
Freedman et al. 2011.	Injection site reactions and flushing in the treatment arm	Back pain, peripheral coldness, and musculoskeletal chest pain	49 SAEs in the MBP8298 arm and 51 in the placebo arm. Most SAEs were considered not related to treatment.	Treatment: AE (8), SAE (8) Placebo: AE (2), SAE (6)
Garren, et al. 2008.	Injection site reactions, CNS***, infections, musculoskeletal**, GIT, psychiatric	Renal and urinary, skin, eye, ear, respiratory, reproductive, investigations (laboratory test, blood pressure, etc.)	9 SAEs reported, but not specifically nominated	0
Goodkin, et al. 2000.	hypertonia, urinary tract infection, headache, and injection site pain	–	Clinical exacerbation (1 case)	0
Kappos, et al. 2000.	Hypersensitivity reactions – study was suspended	paresthesia in the extremities (n = 4), macules on the trunk (n = 1), exanthematous rash (n = 5), dyspnea (n = 2), nausea (n = 2), abdominal pain (n = 1), eosinophilia within a week (n = 2) and hives (n = 4), hypotensive episode (1), syncopal episode (1)	0	10
Karussis, et al. 2012.	Injection site reactions -erythema	–	0	0
van Noort, et al. 2015.	Postural dizziness	GIT pain, nausea, nasopharyngitis, rhinitis, headache, oropharyngolaryngeal pain	0	0
Yadav, et al. 2012.	–	Significant infusion-related adverse events for doses 100 and 200 mg	0	0
Walczak et al. 2013.	Redness and itching	Infections of upper respiratory tracts and lacrimation	0	0
Warren, et al. 2006.	Injection site reactions, facial flushing, mild blood pressure decrease	–	0	0
**Quasi-experimental studies**				
Achiron, et al. 2004.	Redness at the injection site	**–**	0	0
Belogurov, et al. 2016.	Injection site reactions	Rhinitis, general weakness	0	0
Bourdette, et al. 1994.	**–**	Vasovagal rection, vigorous DTH reaction, leukoplastic vasculitis	0	0
Chataway, et al. 2018. a	Injection site reactions	**–**	0	1 (possibly treatment-related allergy)
Chataway, et al. 2018. b	Injection site reactions	Diffuse alopecia, prolonged diarrhea	0	1
Chwojnicki, et al. 2021.	Relapses and the presence of new or enlarging T2 lesions in the CNS (only i.v. administration)	Progression of visual impairment; Liver injury (increased AST and ALT without clinical symptoms, unknown etiology) (only i.v. administration)	0	0
Hohol, et al. 1996.	None reported	None reported	0	0
Ivanova, et al. 2008.	None reported	None reported	0	0
Loftus, et al. 2009.	Injection site reactions	Lymphocytosis, influenza, nasopharyngitis, pain in extremity, dysesthesia, MS relapse, acne, nail disorder, night sweats	Relapse of MS (unlikely related to study vaccine), small intestinal obstruction (unrelated to study vaccine) and pneumonia (unlikely related to study vaccine).	0
Morgan, et al. 2001.	Injection site reactions	**–**	0	0
Pender, et al. 2018.	Transient altered taste (definite); nausea, dizziness, insomnia (possible)	**–**	0	0
Vandenbark, et al. 2008.	None reported	–	0	0
Zhang, et al. 2002.	Injection site reactions	**–**	0	0
Zubizarreta*, et al. 2019.****	**–**	Headache, left leg pain, influenza, cold, palpitations	0	0

*Only those which were considered to be related to the treatment (if not stated otherwise).

**Table cells with ‘-‘signify data which were not reported.

***Not specified in the publication.

****Added data from authors (8 from publication + 5 from extension).

CNS = central nervous system; DTH = Delayed-Type Hypersensitivity; GIT = gastrointestinal; GIT = gastrointestinal; MS = multiple sclerosis.

**Fig 4:**
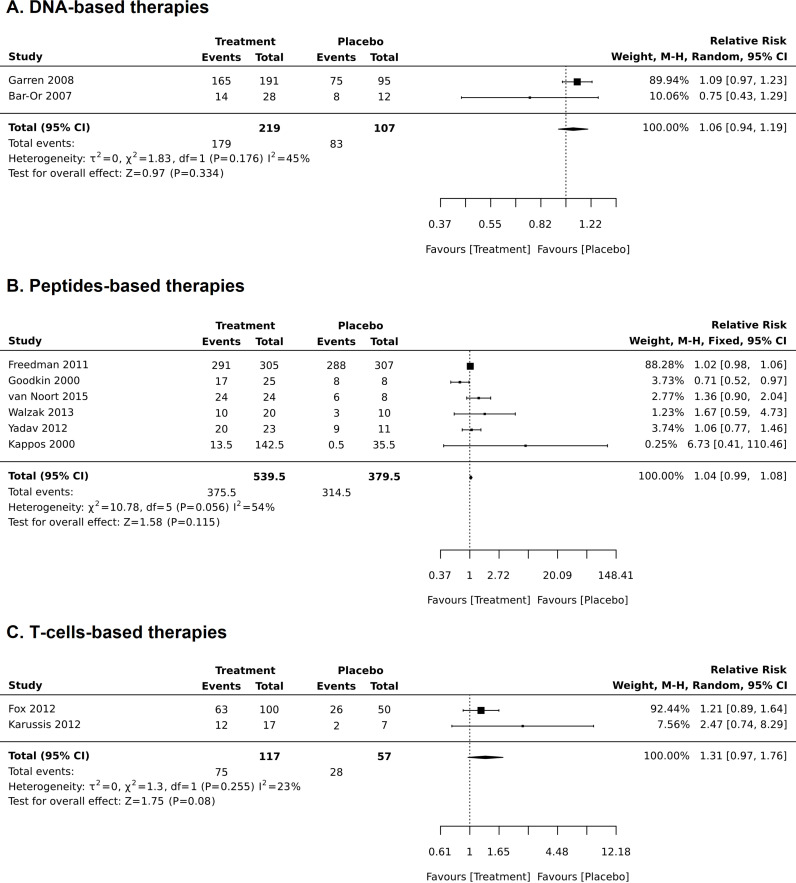
Meta-analysis of the frequency of adverse effects reported in randomized clinical trials.

**Fig 5:**
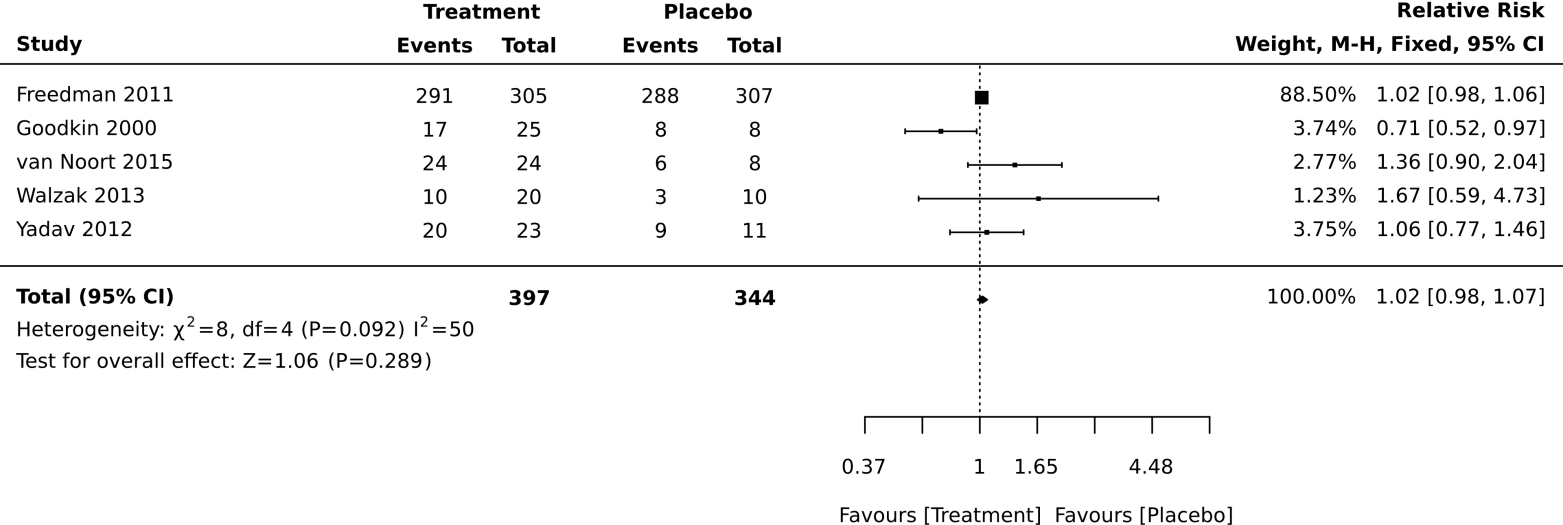
Sensitivity analysis in meta-analysis of the frequency of adverse effects in the peptide-based treatments when a study with low critical appraisal score was omitted.

**Fig 6:**
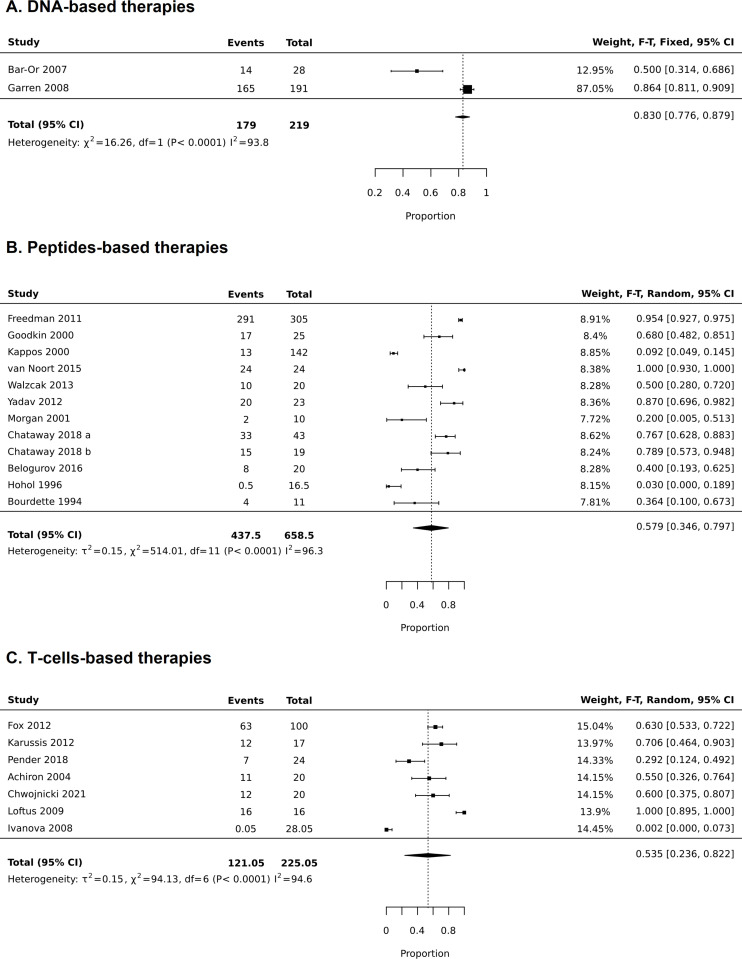
Proportional meta-analysis of the frequency of adverse effects reported in all studies (both randomized clinical trials and quasi-experimental studies).

For RCTs, comparative meta-analyses were performed for both safety outcomes (AEs and SAEs) and included 1418 patients (Figs 4 and 5). For all three types of treatment, there was no statistical difference in occurrence of AEs between the treatment- and placebo-related AEs (for DNA-based treatment RR was 1.06 (0.94–1.10), p = 0.334; for peptides-base treatments RR was 1.04 (0.90–1.08), p = 0.115; for T-cells-based treatments RR was 1.31 (0.97–1.76), p = 0.08), (Fig 4). Heterogeneity for these analyses was moderate (I^2^ was 40–50%), except for T cells-base therapies, where it was low (I^2^ was 23%). The heterogeneity for DNA-based studies was probably mainly due to differences in samples size of the only two studies (14 subjects in Barr-Or et al. [37] and 165 subjects in Garren et al. [41]). Sensitivity analysis was carried out for peptide-based studies based on sample size and critical appraisal score (studies having<50% were excluded). Analysis which included only the small sample or bigger sample size studies exerted higher heterogeneity (results not presented). When a study with low critical appraisal scores was excluded, heterogeneity was slightly decreased (I^2^ was 50%), whereas the results remained similar to the original analysis: RR was 1.03 (0.99–1.08), p = 0.134 (Fig 6).

**Fig 7:**
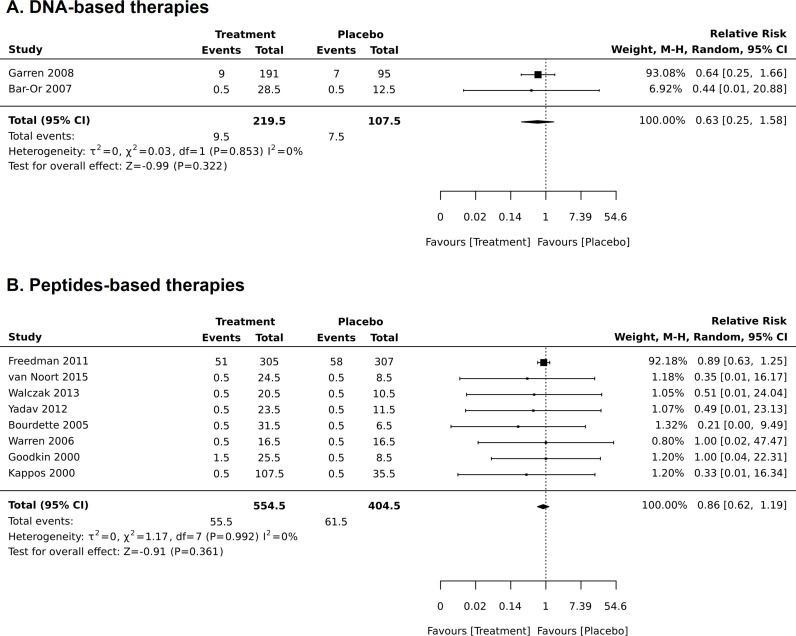
Meta-analysis of the frequency of serious adverse effects reported in randomized clinical trials. Note: study by Freedman et al, 2011. has reported all SAEs, but did not distinguish those which were considered to be related to the treatment from those who were not.

Proportional meta-analysis was executed only for AEs, as there were no reported SAEs in quasi-experimental trials. In total it included 1418 patients from RCTs and 203 from non-RCTs (Fig 7). Tolerogenic dendritic cell-based study (Zubizaretta et al. 2019) [21] was not included in the analysis of AEs in T cell therapies, as the mechanism of this approach is quite different from T cell-based therapies. The occurrence of at least one AEs in DNA-based therapies was 0.83 (0.776–0.879) and was lower for peptide- and T-cell-based treatments: 0.579 (0.346–0.797) and 0.535 (0.236, 0.822), respectively. Almost all studies reported injection site reactions, including redness, pain, and irritation (for more details, see Table 3).

SAEs were reported in a particularly vague manner. Four RCTs reported SAEs. Kappos et al. reported a case of clinical exacerbation [35]; Freedman et al. [36] reported the total number of SAEs (41 in the treatment and 51 in the placebo group), and then stated that the majority of SAEs were not related to the treatment, these SAEs were also not specified; Fox et al. reported 5 SAEs from 5 patients in the treatment groups and 14 SAEs from 9 patients from the placebo group, however, as stated by the authors: “these events were listed as SAEs due to the need of inpatient treatment, but they were not severe in intensity or considered by principal investigators or study physicians to be related to study treatment” [40].

There were no differences in RR for SAEs between the treatments either for DNA-based treatment (RR was 0.63 (0.25–1.58), p = 0.322) or peptide-based treatment (RR was 0.86 (0.62–1.19), p = 0.361) (Fig 5). I^2^ for these meta-analyses was 0. There were no reported SAEs for T cell-based treatments, so meta-analysis for these therapies was not performed (exception is Fox et al. [40], see the explanation above).

It is important to emphasize that the analysis of SAEs for peptides may be influenced by the large number of SAEs reported in the study by Freedman et al [36], where it was not clearly specified how many SAEs were related to the treatment. The authors have reported the total number of SAEs, and then stated that the majority of SAEs were not related to the treatment. As this was a large trial and the only III phase RCT in this review, we have decided to include it in the analysis.

There was one study which reported high occurrence of allergic reactions (9%) and was prematurely suspended [35].

The secondary outcome of this review was to determine the number/proportion of withdrawals from the study due to AEs/SAEs. This is presented in detail in Table 3 for every study. It seems there was a small number of withdrawals from studies, with the exception of the study which was discontinued due to hypersensitivity reactions [35].

We did not find any discrepancies between the protocols (from ClinicalTrials.gov) and reported outcomes in the publications.

SoF according to GRADE approach are presented in Table 4.

**Table 4:** Adverse effects and serious adverse effects in antigen-specific therapies vs. placebo.

Certainty assessment							№ of patients		Effect		Certainty	Importance
№ of studies	**Study design**	**Risk of bias**	**Inconsistency**	**Indirectness**	**Imprecision**	**Other considerations**	**DNA-based therapy**	**Placebo**	**Relative** **(95% CI)**	**Absolute** **(95% CI)**		
Frequency of adverse effects in DNA-based therapies												
2	Randomised trials	Serious^a^	Not serious	Not serious	Serious^b,c^	None	179/219 (81.7%)	93/107 (86.9%)	**RR 1.06** (0.94 to 1.19)	**52 more per 1,000** (from 52 fewer to 165 more)	⨁⨁○○ Low	
Frequency of serious adverse effects in DNA-based therapies												
2	Randomised trials	Serious^a^	Not serious	Not serious	Very serious^b,c,d^	None	9.5/219.5 (4.3%)	7.5/107.5 (7.0%)	**RR 0.63** (0.25 to 1.58)	**26 fewer per 1,000** (from 52 fewer to 40 more)	⨁○○○ Very low	
Frequency of adverse effects in peptide-based therapies												
6	Randomised trials	Not serious^e^	Serious^f^	Not serious	Not serious	None	375.5/539.5 (69.6%)	314.5/379.5 (82.9%)	**RR 1.04** (0.99 to 1.08)	**33 more per 1,000** (from 8 fewer to 66 more)	⨁⨁⨁○ Moderate	
Frequency of serious adverse effects in peptide-based therapies												
8	Randomised trials	Very serious^g,h^	Not serious	Not serious	Serious^c^	None	55.5/554.5 (10.0%)	61.5/404.5 (15.2%)	**RR 0.86** (0.62 to 1.19)	**21 fewer per 1,000** (from 58 fewer to 29 more)	⨁○○○ Very low	
Frequency of adverse effects in T cell-based therapies												
2	Randomised trials	Serious^i^	Not serious	Not serious	Serious^c^	None	75/117 (64.1%)	28/57 (49.1%)	**RR 1.31** (0.97 to 1.76)	**152 more per 1,000** (from 15 fewer to 373 more)	⨁⨁○○ Low	

aRandomization and blinding of the participants are uncertain (both studies) plus blinding of the staff was not performed in one study.

bWide confident intervals.

cLess than 300 events.

dSAEs in Garren et al. were not named.

eConcerns are randomization (uncertain in 50%) and blinding in staff (67%).

fModerate to high heterogeneity.

gConcerns are randomization (uncertain in 62.5%) and blinding in staff (75%).

hA study by Friedman et al reported “49 treatment-emergent SAEs in the MBP8298 arm and 51 in the placebo arms. Most SAEs were considered not related to treatment.”

iRandomization and blinding of the participants are uncertain (both studies) plus blinding of the stuaf was not performed in one study and uncertain in the other study.

CI = confidence interval; RR = Risk ratio.

## Discussion

This systematic review has provided an extensive overview of antigen-specific therapies in MS, including products based on technologies such as T cells, peptides, and DNA. These novel treatments appear to be safe. There were no significant differences in the occurrence of AEs and SAEs in intervention compared to the placebo groups and there was a low frequency of SAEs in MS patients during the treatment. The most frequent AEs were local reactions to injections, such as redness, erythema, pain. These results should be interpreted with caution due to small sample sizes of early-stage clinical trials, the rareness of events, and lack of blinding in many studies. Currently, there is no sufficient data to make a conclusion on the efficacy of antigen-specific therapies. It should be borne in mind that the majority of the reviewed studies were phase I or II clinical trials and therefore were not powered nor designed to evaluate efficacy as primary outcome.

We have not found any systematic review dealing with the same topic of safety and efficacy of antigen-specific therapies in MS patients, therefore, it is difficult to comment on our results in the context of previous research. A recent systematic review and meta-analysis of tolerance-inducing cell products in patients with autoimmune diseases or receiving organ transplantation has found that these products are safe, although the quality of data was in risk of moderate to severe bias in non-RCTs and moderate risk in RCTs [60]. This review has analyzed cell products, including dendritic cells, regulatory T cells and mesenchymal stem cells and included patients who underwent organ transplantation, as well as those with autoimmune diseases, including MS, diabetes mellitus type 1, Crohn’s disease, and rheumatoid arthritis.

This review has included highly heterogeneous studies in terms of patients (having RR or progressive MS forms), route of administration (p.o., i.v., s.c., i.m., t.d., i.d.), type of study (RCT or quasi-experimental), length of treatment and follow up (one day to 8 years), administration frequency (one day to 3 month-interval), number of patents (10 to 612), and therapies (various targets within 3 main groups of therapies: peptides, cells, DNA). Additionally, there was a remarkable variability in outcomes reported regarding efficacy: scales for MS disability assessment, various MRI endpoints, relapse rate/time to relapse. Even when the same scale (EDSS) was used across the studies, reporting of its measurements was heterogenous, as some studies reported mean, or median values for all patients in trials, whereas others have reported the number of patients who improved/remained stable/deteriorated. These drawbacks have precluded the quantitative analyses of the therapies’ efficacy; instead we have provided a narrative analysis in the Results section. In the previously mentioned systematic review evaluating cell-based therapies inducing tolerance, the clinical response in the intervention group was at least equal to the control group, or slightly higher [60]. Based on the results of the current review, significant clinical benefits with DNA vaccines were not observed neither by disability scales, nor by MRI. It seems that the concept of DNA vaccines has been abandoned in MS therapy, so recent and currently ongoing trials assess other modalities, such as peptides- and particularly cell-based products. Among these therapies, there were different targets, mostly these were different autoantigens (MOG, PLP, MBP, sections of TCR or whole TCR). Interestingly, there are promising two trials (one is ongoing), evaluating specific T cells against EBV. This concept emerged from the growing body of evidence that EBV triggers development of MS, as infection with EBV increases the risk more than 30-fold [61].

Safety assessment was an important outcome of this review. There was a low occurrence of SAEs and no differences in frequency of AEs/SAEs between intervention and placebo groups. These findings are in line with previously mentioned systematic review evaluating cell-based therapies inducing tolerance [60]. Still, it has to be emphasized that the certainty for these results was very low for SAEs in peptide- and DNA-based therapies, whereas it was low for AEs in DNA- and T cells-based therapies and moderate for AEs in peptide-based therapies (Table 4). According to the proportional meta-analysis of AEs, it appears that there is a greater risk of occurrence of AEs after treatment with DNA-based products (0.83) compared to the T-cells-based (0.535) and peptides-based products (0.579). Due to lack of information, we could not distinguish AEs into different grades based on severity. As expected, the most frequent AEs were injection reactions.

The quality of the studies included in this review varied highly. For quasi-experimental studies, critical appraisal scores were in the range 45% to 78%. All studies from this group included one-group pretest-posttest design. The absence of a control group hinders the ability to conclusively determine the relationship between AEs/SAEs and the administered therapy. In such instances, the link between an AEs/SAEs and the intervention relies on the investigators’ judgment. Additionally, multiple measurements of the outcome both pre and post the intervention/exposure lacked in all studies. Moreover, in 73% of the studies follow up was incomplete, mostly because the authors failed to report the reasons for loss to follow up. Another important potential confounding factor in these studies was the simultaneous use of immunosuppressive therapies in participants, in approximately half of the reviewed studies. Regarding RCTs, in as many as 75% of them, the primary sources of bias were the absence of blinding and randomization. Failure to randomize participants can introduce selection bias. Furthermore, awareness of which group participants belong to in the trial can influence their behavior and how they respond to subjective outcome measures.

MS exhibits various clinical courses, each responding differently to treatments. Presently, there are available drugs that effectively target the inflammatory aspect of the disease, particularly in RRMS, leading to positive clinical outcomes. However, the more severe forms of MS, namely PPMS and SPMS, have limited therapeutic options. Despite affecting fewer MS patients, PPMS and SPMS individuals are often included in research studies due to the scarcity of treatment options for these forms. Given that RRMS generally exhibits a more favorable response to treatment, it is anticipated that research outcomes would demonstrate better results in RRMS patients compared to those with PPMS and SPMS. However, the analyzed population in this review lacks a sufficient number of patients with PPMS or RRMS, preventing a meaningful comparison of treatment efficacy across different clinical courses. This underscores the necessity for clinical trials specifically designed to determine which clinical course exhibits a more favorable response to antigen-specific treatment, aiming to provide more robust and definitive conclusions.

The limitation of this review is that it relies mostly on published studies, which can lead to overestimating effects if unpublished negative or null-result studies are missing. Namely, a substantial number of studies were still officially ongoing despite their estimated completion dates having passed, or they had been completed but had not posted their results. We contacted all responsible researchers to inquire about the study outcomes; however, we did not receive any responses. Moreover, it is debatable whether the different T cell therapies (e.g., ones targeting EBV, Treg cells, myelin-reactive T cells) should be grouped together in the meta-analyses for safety outcomes, given the varied mechanisms of actions. However, we chose to keep T cell-based therapy grouped as one modality, as these therapies share common features, including cellular therapy techniques, T cell expansion and modulation strategies, and a highly personalized approach to immune system modulation. In this way, we aimed to provide a clearer understanding of the safety profiles restricted to T cells-based antigen specific therapies.

In conclusion, this systematic review has provided an extensive overview of different antigen-specific tolerance-inducing therapies in MS. There were no significant differences in the occurrence of AEs and SAEs in intervention compared to the placebo groups and there was a low frequency of SAEs in MS patients during the treatment. Currently, there is no sufficient data to make a conclusion on the efficacy of antigen-specific therapies. It should be kept in mind that the majority of the reviewed studies were phase I or II clinical trials, and that larger, well-designed studies with high quality are needed to ensure ultimate conclusions.

## Supporting information

S1 Appendix Search strategies for databases. (DOCX)

S2 Appendix Searches conducted on 5.2.2024. (DOCX)

S1 Table List of all included and excluded studies after full text screening. (DOCX)

S1 Checklist PRISMA 2020 checklist. (DOCX)
