# Supplementary material for: Safety and efficacy of antigen-specific therapeutic approaches for multiple sclerosis: Systematic review
Source: PLoS One. 2025 May 19;20(5):e0320814. doi: 10.1371/journal.pone.0320814 (PMC12088042; doi:10.1371/journal.pone.0320814)
Supplement: S1 Appendix — (DOCX) [file pone.0320814.s001.docx]

### **S1 Appendix: Search strategies for databases**

## **PubMed**

## Search conducted on 26.08.2021 (21:33) and 489 records retrieved at this time point.

1. (antigen-specific[tw] OR tolerance inducing[tw] OR tolerogenic[tw] OR immune tolerance[tw] OR immune regulation[tw] OR immunotherapy[tw] OR antigen treatment[tw] OR myelin basic protein[tw] OR myelin peptide*[tw] OR myelin antigen[tw] OR DNA vaccine*[tw] OR T cell vaccine[tw] OR T-cell vaccine*[tw] OR recombinant T-cell[tw] OR peptide based[tw] OR protein based[tw] OR cell-based[tw] OR dendritic cell[tw] OR tolerogenic dendritic cell*[tw] OR peptide-loaded[tw] OR antigen-specific tolerance*[tw] OR DNA encoding[tw] OR peptide-coupled[tw] OR T-cell receptor[tw] OR ligand[tw] OR RTL therapy[tw] OR MBP[tw] OR altered peptide ligand[tw])
2. (multiple sclerosis[tw] OR MS[tw] OR relapsing remitting multiple sclerosis[tw] OR secondary progressive multiple sclerosis[tw] OR primary progressive multiple sclerosis[tw])
3. 1 AND 2
4. (clinical trial[tw] OR phase 1[tw] OR phase I[tw] OR phase 2[tw] OR phase-3[tw] OR phase-4[tw] OR phase II[tw] OR phase III[tw] OR phase IV[tw] OR phase 1a[tw] OR phase Ia[tw] OR phase 2a[tw] OR phase-IIa[tw] OR phase 1b[tw] OR phase Ib[tw] OR phase 2b[tw] OR phase-IIb[tw] OR preliminary study[tw] OR clinical study[tw] OR first-in-human[tw] OR first-in-man[tw] OR randomized*[tw] OR non-randomized*[tw])
5. 3 AND 4

(antigen-specific[tw] OR tolerance inducing[tw] OR tolerogenic[tw] OR immune tolerance[tw] OR immune regulation[tw] OR immunotherapy[tw] OR antigen treatment[tw] OR myelin basic protein[tw] OR myelin peptide*[tw] OR myelin antigen[tw] OR DNA vaccine*[tw] OR T cell vaccine[tw] OR T-cell vaccine*[tw] OR recombinant T-cell[tw] OR peptide based[tw] OR protein based[tw] OR cell-based[tw] OR dendritic cell[tw] OR tolerogenic dendritic cell*[tw] OR peptide-loaded[tw] OR antigen-specific tolerance*[tw] OR DNA encoding[tw] OR peptide-coupled[tw] OR T-cell receptor[tw] OR ligand[tw] OR RTL therapy[tw] OR MBP[tw] OR altered peptide ligand[tw]) AND (multiple sclerosis[tw] OR MS[tw] OR relapsing remitting multiple sclerosis[tw] OR secondary progressive multiple sclerosis[tw] OR primary progressive multiple sclerosis[tw]) AND (clinical trial[tw] OR phase 1[tw] OR phase I[tw] OR phase 2[tw] OR phase-3[tw] OR phase-4[tw] OR phase II[tw] OR phase III[tw] OR phase IV[tw] OR phase 1a[tw] OR phase Ia[tw] OR phase 2a[tw] OR phase-IIa[tw] OR phase 1b[tw] OR phase Ib[tw] OR phase 2b[tw] OR phase-IIb[tw] OR preliminary study[tw] OR clinical study[tw] OR first-in-human[tw] OR first-in-man[tw] OR randomized*[tw] OR non-randomized*[tw])

**2- Web of Science**

Search conducted on 31.08.2021 (13.48). 837 records retrieved at this time point.

1. myelin OR ligand OR antigen OR epitope OR tolerogenic OR tolerance OR immunotherapy OR DNA or T cell OR dendritic cell OR peptide OR protein OR cell based OR RTL OR TCR OR altered peptide ligand OR antigen-coupled (Title)
2. multiple sclerosis OR relapsing remitting multiple sclerosis OR secondary progressive multiple sclerosis OR primary progressive multiple sclerosis (Title)
3. 1 AND 2
4. clinical study OR clinical trial OR phase OR first-in-human OR first-in-man OR trial OR human study (Topic)
5. English (Language)
6. Book Chapter OR Book Review OR Review (Document Type)
7. 3 AND 4 AND 5 NOT 6

multiple sclerosis OR relapsing remitting multiple sclerosis OR secondary progressive multiple sclerosis OR primary progressive multiple sclerosis (Title) and clinical study OR clinical trial OR phase OR first-in-human OR first-in-man OR trial OR human study (Topic) and myelin OR ligand OR antigen OR epitope OR tolerogenic OR tolerance OR immunotherapy OR DNA or T cell OR dendritic cell OR peptide OR protein OR cell based OR RTL OR TCR OR altered peptide ligand OR antigen-coupled (Title) and English (Language) not Book Chapter OR Book Review OR Review (Document Type)

## **3- CINAHL – EBSCO**

974 records were found on 26.08.2021 (14.06).  900 of 974 records were accessible

1. TI ( myelin OR ligand OR antigen OR 'antigen specific' OR epitope OR tolerogenic OR tolerance OR immunotherapy OR DNA or 'T cell' OR 'dendritic cell' OR peptide OR protein OR 'cell based' OR RTL OR TCR OR 'altered peptide ligand' OR 'antigen-coupled' )
2. TI ( ‘multiple sclerosis’ or ‘ms’ or ‘progressive multiple sclerosis’ or ‘relapsing remitting’ )
3. 1AND 2
4. AB ( 'clinical study' OR 'clinical trial' OR phase OR 'first-in-human' OR 'first-in-man' OR trial )
5. TI ( Review OR 'Systematic Review' OR 'Book Chapter' OR 'meta-analysis' )
6. TI ( 'murine model' OR EAE OR 'cuprizone model' OR 'animal model' OR 'animal study' Or mice OR mouse Or rat OR 'in vitro' OR 'in vivo' )
7. 3 AND 4 NOT 5 NOT 6

TI ( ‘multiple sclerosis’ or ‘ms’ or ‘progressive multiple sclerosis’ or ‘relapsing remitting’ ) AND AB ( 'clinical study' OR 'clinical trial' OR phase OR 'first-in-human' OR 'first-in-man' OR trial ) AND TI ( myelin OR ligand OR antigen OR 'antigen specific' OR epitope OR tolerogenic OR tolerance OR immunotherapy OR DNA or 'T cell' OR 'dendritic cell' OR peptide OR protein OR 'cell based' OR RTL OR TCR OR 'altered peptide ligand' OR 'antigen-coupled' ) NOT TI ( Review OR 'Systematic Review' OR 'Book Chapter' OR 'meta-analysis' ) NOT TI ( 'murine model' OR EAE OR 'cuprizone model' OR 'animal model' OR 'animal study' Or mice OR mouse Or rat OR 'in vitro' OR 'in vivo' )

## **4- Cochrane**

418 records were found on 26.08.2021 14:47.

1. (''antigen-specific'' OR ''tolerance inducing'' OR tolerogenic OR ''immune tolerance'' OR ''antigen treatment'' OR myelin OR ''DNA vaccine'' OR ''T cell vaccine'' OR ''peptide based'' OR ''cell based'' OR ''dendritic based'' OR ''dendritic cell'' OR RTL OR TCR OR ''altered peptide ligand'') in Title Abstract Keyword
2. (''multiple sclerosis'' or ms or ''relapsing remitting multiple sclerosis'' or ''progressive multiple sclerosis'') in Title Abstract Keyword
3. 1 AND 2
4. (''clinical trial'' OR phase OR ''clinical study'' OR first-in-human OR first-in-man OR trial in Publication Type NOT review or ''systematic review'' or ''meta analysis'' or ''book chapter'') in Publication Type
5. 3 AND 4

''multiple sclerosis'' or ms or ''relapsing remitting multiple sclerosis'' or ''progressive multiple sclerosis'' in Title Abstract Keyword AND ''antigen-specific'' OR ''tolerance inducing'' OR tolerogenic OR ''immune tolerance'' OR ''antigen treatment'' OR myelin OR ''DNA vaccine'' OR ''T cell vaccine'' OR ''peptide based'' OR ''cell based'' OR ''dendritic based'' OR ''dendritic cell'' OR RTL OR TCR OR ''altered peptide ligand'' in Title Abstract Keyword AND ''clinical trial'' OR phase OR ''clinical study'' OR first-in-human OR first-in-man OR trial in Publication Type NOT review or ''systematic review'' or ''meta analysis'' or ''book chapter'' in Publication Type - (Word variations have been searched)

## **5- ICTRP WHO**

43 results were found on 31.08.2021 (22:07).

Recruitment status is set to **ALL**

Filter based on Title using:

multiple sclerosis OR relapsing remitting multiple sclerosis OR secondary progressive multiple sclerosis OR primary progressive multiple sclerosis

Filter based on Intervention using:

myelin OR tolerance OR tolerogenic OR MOG OR MBP OR epitope OR peptide OR antigen OR DNA OR T cell OR peptide based OR cell-based OR dendritic OR peptide loaded OR RTL OR TCR OR altered peptide ligand OR antigen coupled OR immunotherapy

## **6 - clinicaltrials.gov**

230 studies were found on 31.08.2021 (16:30).

myelin OR tolerance OR tolerogenic OR MOG OR MBP OR epitope OR peptide OR antigen OR DNA OR T cell OR peptide based OR cell-based OR dendritic OR peptide-loaded OR RTL OR TCR OR altered peptide ligand OR antigen coupled OR immunotherapy | Interventional Studies | Multiple Sclerosis | Adult
