# Supplementary material for: Safety and efficacy of antigen-specific therapeutic approaches for multiple sclerosis: Systematic review
Source: PLoS One. 2025 May 19;20(5):e0320814. doi: 10.1371/journal.pone.0320814 (PMC12088042; doi:10.1371/journal.pone.0320814)
Supplement: S2 Appendix — (DOCX) [file pone.0320814.s002.docx]

S2 Appendix :Searches conducted on 5.2.2024.

1. **Pubmed**

| Search | Query | Records retrieved |
| --- | --- | --- |
| #1 | (antigen-specific[tw] OR tolerance inducing[tw] OR tolerogenic[tw] OR immune tolerance[tw] OR immune regulation[tw] OR immunotherapy[tw] OR antigen treatment[tw] OR myelin basic protein[tw] OR myelin peptide*[tw] OR myelin antigen[tw] OR DNA vaccine*[tw] OR T cell vaccine[tw] OR T-cell vaccine*[tw] OR recombinant T-cell[tw] OR peptide based[tw] OR protein based[tw] OR cell-based[tw] OR dendritic cell[tw] OR tolerogenic dendritic cell*[tw] OR peptide-loaded[tw] OR antigen-specific tolerance*[tw] OR DNA encoding[tw] OR peptide-coupled[tw] OR T-cell receptor[tw] OR ligand[tw] OR RTL therapy[tw] OR MBP[tw] OR altered peptide ligand[tw]) | 54,958 |
| #2 | (multiple sclerosis[tw] OR MS[tw] OR relapsing remitting multiple sclerosis[tw] OR secondary progressive multiple sclerosis[tw] OR primary progressive multiple sclerosis[tw]) | 39,766 |
| #3 | (antigen-specific[tw] OR tolerance inducing[tw] OR tolerogenic[tw] OR immune tolerance[tw] OR immune regulation[tw] OR immunotherapy[tw] OR antigen treatment[tw] OR myelin basic protein[tw] OR myelin peptide*[tw] OR myelin antigen[tw] OR DNA vaccine*[tw] OR T cell vaccine[tw] OR T-cell vaccine*[tw] OR recombinant T-cell[tw] OR peptide based[tw] OR protein based[tw] OR cell-based[tw] OR dendritic cell[tw] OR tolerogenic dendritic cell*[tw] OR peptide-loaded[tw] OR antigen-specific tolerance*[tw] OR DNA encoding[tw] OR peptide-coupled[tw] OR T-cell receptor[tw] OR ligand[tw] OR RTL therapy[tw] OR MBP[tw] OR altered peptide ligand[tw]) AND (multiple sclerosis[tw] OR MS[tw] OR relapsing remitting multiple sclerosis[tw] OR secondary progressive multiple sclerosis[tw] OR primary progressive multiple sclerosis[tw]) | 1,022 |
| #4 | (clinical trial[tw] OR phase 1[tw] OR phase I[tw] OR phase 2[tw] OR phase-3[tw] OR phase-4[tw] OR phase II[tw] OR phase III[tw] OR phase IV[tw] OR phase 1a[tw] OR phase Ia[tw] OR phase 2a[tw] OR phase-IIa[tw] OR phase 1b[tw] OR phase Ib[tw] OR phase 2b[tw] OR phase-IIb[tw] OR preliminary study[tw] OR clinical study[tw] OR first-in-human[tw] OR first-in-man[tw] OR randomized*[tw] OR non-randomized*[tw]) | 197,619 |
| #5 | (antigen-specific[tw] OR tolerance inducing[tw] OR tolerogenic[tw] OR immune tolerance[tw] OR immune regulation[tw] OR immunotherapy[tw] OR antigen treatment[tw] OR myelin basic protein[tw] OR myelin peptide*[tw] OR myelin antigen[tw] OR DNA vaccine*[tw] OR T cell vaccine[tw] OR T-cell vaccine*[tw] OR recombinant T-cell[tw] OR peptide based[tw] OR protein based[tw] OR cell-based[tw] OR dendritic cell[tw] OR tolerogenic dendritic cell*[tw] OR peptide-loaded[tw] OR antigen-specific tolerance*[tw] OR DNA encoding[tw] OR peptide-coupled[tw] OR T-cell receptor[tw] OR ligand[tw] OR RTL therapy[tw] OR MBP[tw] OR altered peptide ligand[tw]) AND (multiple sclerosis[tw] OR MS[tw] OR relapsing remitting multiple sclerosis[tw] OR secondary progressive multiple sclerosis[tw] OR primary progressive multiple sclerosis[tw]) AND (clinical trial[tw] OR phase 1[tw] OR phase I[tw] OR phase 2[tw] OR phase-3[tw] OR phase-4[tw] OR phase II[tw] OR phase III[tw] OR phase IV[tw] OR phase 1a[tw] OR phase Ia[tw] OR phase 2a[tw] OR phase-IIa[tw] OR phase 1b[tw] OR phase Ib[tw] OR phase 2b[tw] OR phase-IIb[tw] OR preliminary study[tw] OR clinical study[tw] OR first-in-human[tw] OR first-in-man[tw] OR randomized*[tw] OR non-randomized*[tw]) | 53 |

1. **Web of Science**

| Search | Query | Records retrieved |
| --- | --- | --- |
| #1 | myelin OR ligand OR antigen OR epitope OR tolerogenic OR tolerance OR immunotherapy OR DNA or T cell OR dendritic cell OR peptide OR protein OR cell based OR RTL OR TCR OR altered peptide ligand OR antigen-coupled (Title) | 216,578 |
| #2 | multiple sclerosis OR relapsing remitting multiple sclerosis OR secondary progressive multiple sclerosis OR primary progressive multiple sclerosis (Title) | 12,060 |
| #3 | (myelin OR ligand OR antigen OR epitope OR tolerogenic OR tolerance OR immunotherapy OR DNA or T cell OR dendritic cell OR peptide OR protein OR cell based OR RTL OR TCR OR altered peptide ligand OR antigen-coupled) AND (multiple sclerosis OR relapsing remitting multiple sclerosis OR secondary progressive multiple sclerosis OR primary progressive multiple sclerosis) (Title) | 521 |
| #4 | clinical study OR clinical trial OR phase OR first-in-human OR first-in-man OR trial OR human study (Topic) | 1,738 |
| #5 | (multiple sclerosis OR relapsing remitting multiple sclerosis OR secondary progressive multiple sclerosis OR primary progressive multiple sclerosis (Title)) and (clinical study OR clinical trial OR phase OR first-in-human OR first-in-man OR trial OR human study (Topic)) and (myelin OR ligand OR antigen OR epitope OR tolerogenic OR tolerance OR immunotherapy OR DNA or T cell OR dendritic cell OR peptide OR protein OR cell based OR RTL OR TCR OR altered peptide ligand OR antigen-coupled (Title)) and (English (Language)) not (Book Chapter OR Book Review OR Review (Document Type)) | 139 |

1. **CINAHL – EBSCO**

| Search | Query | Records retrieved |
| --- | --- | --- |
| #1 | TI ( myelin OR ligand OR antigen OR 'antigen specific' OR epitope OR tolerogenic OR tolerance OR immunotherapy OR DNA or 'T cell' OR 'dendritic cell' OR peptide OR protein OR 'cell based' OR RTL OR TCR OR 'altered peptide ligand' OR 'antigen-coupled' ) | 142 |
| #2 | TI ( ‘multiple sclerosis’ or ‘ms’ or ‘progressive multiple sclerosis’ or ‘relapsing remitting’ ) | 4,559 |
| #3 | TI ( myelin OR ligand OR antigen OR 'antigen specific' OR epitope OR tolerogenic OR tolerance OR immunotherapy OR DNA or 'T cell' OR 'dendritic cell' OR peptide OR protein OR 'cell based' OR RTL OR TCR OR 'altered peptide ligand' OR 'antigen-coupled' ) AND TI ( ‘multiple sclerosis’ or ‘ms’ or ‘progressive multiple sclerosis’ or ‘relapsing remitting’ ) | 164 |
| #4 | AB ( 'clinical study' OR 'clinical trial' OR phase OR 'first-in-human' OR 'first-in-man' OR trial ) | 60,598 |
| #5 | TI ( Review OR 'Systematic Review' OR 'Book Chapter' OR 'meta-analysis' ) | 2,112 |
| #6 | TI ( 'murine model' OR EAE OR 'cuprizone model' OR 'animal model' OR 'animal study' Or mice OR mouse Or rat OR 'in vitro' OR 'in vivo' ) | 42,216 |
| #7 | TI ( ‘multiple sclerosis’ or ‘ms’ or ‘progressive multiple sclerosis’ or ‘relapsing remitting’ ) AND AB ( 'clinical study' OR 'clinical trial' OR phase OR 'first-in-human' OR 'first-in-man' OR trial ) AND TI ( myelin OR ligand OR antigen OR 'antigen specific' OR epitope OR tolerogenic OR tolerance OR immunotherapy OR DNA or 'T cell' OR 'dendritic cell' OR peptide OR protein OR 'cell based' OR RTL OR TCR OR 'altered peptide ligand' OR 'antigen-coupled' ) NOT TI ( Review OR 'Systematic Review' OR 'Book Chapter' OR 'meta-analysis' ) NOT TI ( 'murine model' OR EAE OR 'cuprizone model' OR 'animal model' OR 'animal study' Or mice OR mouse Or rat OR 'in vitro' OR 'in vivo' ) | 125 |

1. **Cochrane**

| Search | Query | Records retrieved |
| --- | --- | --- |
| #1 | (''antigen-specific'' OR ''tolerance inducing'' OR tolerogenic OR ''immune tolerance'' OR ''antigen treatment'' OR myelin OR ''DNA vaccine'' OR ''T cell vaccine'' OR ''peptide based'' OR ''cell based'' OR ''dendritic based'' OR ''dendritic cell'' OR RTL OR TCR OR ''altered peptide ligand'') in Title Abstract Keyword | 14007 |
| #2 | (''multiple sclerosis'' or ms or ''relapsing remitting multiple sclerosis'' or ''progressive multiple sclerosis'') | 4804 |
| #3 | (''antigen-specific'' OR ''tolerance inducing'' OR tolerogenic OR ''immune tolerance'' OR ''antigen treatment'' OR myelin OR ''DNA vaccine'' OR ''T cell vaccine'' OR ''peptide based'' OR ''cell based'' OR ''dendritic based'' OR ''dendritic cell'' OR RTL OR TCR OR ''altered peptide ligand'') AND (''multiple sclerosis'' or ms or ''relapsing remitting multiple sclerosis'' or ''progressive multiple sclerosis'') in Title Abstract Keyword | 314 |
| #4 | (''clinical trial'' OR phase OR ''clinical study'' OR first-in-human OR first-in-man OR trial NOT review or ''systematic review'' or ''meta analysis'' or ''book chapter'') in Publication type | 118126 |
| #5 | (''antigen-specific'' OR ''tolerance inducing'' OR tolerogenic OR ''immune tolerance'' OR ''antigen treatment'' OR myelin OR ''DNA vaccine'' OR ''T cell vaccine'' OR ''peptide based'' OR ''cell based'' OR ''dendritic based'' OR ''dendritic cell'' OR RTL OR TCR OR ''altered peptide ligand'') AND (''multiple sclerosis'' or ms or ''relapsing remitting multiple sclerosis'' or ''progressive multiple sclerosis'') in Title Abstract Keyword AND (''clinical trial'' OR phase OR ''clinical study'' OR first-in-human OR first-in-man OR trial NOT review or ''systematic review'' or ''meta analysis'' or ''book chapter'') in Publication type | 84 |

1. **ICTRP WHO**

| Search | Query | Records retrieved |
| --- | --- | --- |
| #1 | multiple sclerosis OR relapsing remitting multiple sclerosis OR secondary progressive multiple sclerosis OR primary progressive multiple sclerosis (Filter based on Title) | 701 |
| #2 | multiple sclerosis OR relapsing remitting multiple sclerosis OR secondary progressive multiple sclerosis OR primary progressive multiple sclerosis (Filter based on Title) AND myelin OR tolerance OR tolerogenic OR MOG OR MBP OR epitope OR peptide OR antigen OR DNA OR T cell OR peptide based OR cell-based OR dendritic OR peptide loaded OR RTL OR TCR OR altered peptide ligand OR antigen coupled OR immunotherapy (Filter based on Intervention) | 12 |

1. **clinicaltrials.gov**

| Search | Query | Records retrieved |
| --- | --- | --- |
| #1 | myelin OR tolerance OR tolerogenic OR MOG OR MBP OR epitope OR peptide OR antigen OR DNA OR T cell OR peptide based OR cell-based OR dendritic OR peptide-loaded OR RTL OR TCR OR altered peptide ligand OR antigen coupled OR immunotherapy \| Interventional Studies \| Multiple Sclerosis \| Adult | 10 |

# **List of articles excluded with reasons**

| Study | Reason of exclusion |
| --- | --- |
| **NCT05417269** IMCY-0141 Safety and Efficacy in Multiple Sclerosis - ISEMIS Study | Ongoing study |
| **EUCTR2022-000801-28-DE** Peptide-coupled Red Blood Cells for the Treatment of Multiple Sclerosis | Ongoing study |
| **NCT05080270** Feasibility Study of Tolerogenic Fibroblasts in Patients With Refractory Multiple Sclerosis (MSFibroblast) | Results not posted |
