# Supplementary material for: Safety and efficacy of antigen-specific therapeutic approaches for multiple sclerosis: Systematic review
Source: PLoS One. 2025 May 19;20(5):e0320814. doi: 10.1371/journal.pone.0320814 (PMC12088042; doi:10.1371/journal.pone.0320814)
Supplement: S1 Table — (DOCX) [file pone.0320814.s003.docx]

S1 Table. List of all included and excluded studies after full text screening

| **Included studies** | | |
| --- | --- | --- |
| **Study** | **Name of the study** | **Date of extraction** |
| 1 | Belogurov A, Zakharov K, Lomakin Y, Surkov K, Avtushenko S, Kruglyakov P, et al. CD206-targeted liposomal myelin basic protein peptides in patients with multiple sclerosis resistant to first-line disease-modifying therapies: a first-in-human, proof-of-concept dose-escalation study. Neurotherapeutics. 2016;13:895-904. | November 2021 |
| 2 | Bourdette DN, Whitham RH, Chou YK, Morrison WJ, Atherton J, Kenny C, et al. Immunity to TCR peptides in multiple sclerosis. I. Successful immunization of patients with synthetic V beta 5.2 and V beta 6.1 CDR2 peptides. Journal of immunology (Baltimore, Md: 1950). 1994;152(5):2510-9 | November 2021 |
| 3 | Chataway J, Martin K, Barrell K, Sharrack B, Stolt P, Wraith DC. Effects of ATX-MS-1467 immunotherapy over 16 weeks in relapsing multiple sclerosis. Neurology. 2018;90(11):e955-e62 | November 2021 |
| 4 | Hohol MJ, Khoury SJ, Cook SL, Orav EJ, Hafler DA, Weiner HL. Three‐year open protocol continuation study of oral tolerization with myelin antigens in multiple sclerosis and design of a phase III pivotal trial. Annals of the New York Academy of Sciences. 1996;778(1):243-50. | November 2021 |
| 5 | Morgan EE, Nardo CJ, Diveley JP, Kunin J, Bartholomew RM, Moss RB, et al. Vaccination with a CDR2 BV6S2/6S5 peptide in adjuvant induces peptide‐specific T‐cell responses in patients with multiple sclerosis. Journal of Neuroscience Research. 2001;64(3):298-301. | November 2021 |
| 6 | Vandenbark AA, Culbertson NE, Bartholomew RM, Huan J, Agotsch M, LaTocha D, et al. Therapeutic vaccination with a trivalent T‐cell receptor (TCR) peptide vaccine restores deficient FoxP3 expression and TCR recognition in subjects with multiple sclerosis. Immunology. 2008;123(1):66-78. | November 2021 |
| 7 | Achiron A, Lavie G, Kishner I, Stern Y, Sarova-Pinhas I, Ben-Aharon T, et al. T cell vaccination in multiple sclerosis relapsing–remitting nonresponders patients. Clinical immunology. 2004;113(2):155-60. | December 2021 |
| 8 | Bar-Or A, Pender M, Hodgkinson S, Broadley S, Lindsey J, Ioannides Z, et al. Phase I open-label extension and imaging data for ATA188, an allogeneic Epstein-Barr virus-targeted multiple sclerosis immunotherapy. 2021, link: https://www.charcot-ms.org/files/30_ECF2021_Poster_CL_Bar-Or_A.pdf | December 2021 |
| 9 | Chwojnicki K, Iwaszkiewicz-Grześ D, Jankowska A, Zieliński M, Łowiec P, Gliwiński M, et al. Administration of CD4+ CD25 high CD127− FoxP3+ Regulatory T Cells for Relapsing-Remitting Multiple Sclerosis: A Phase 1 Study. BioDrugs. 2021;35:47-60. | December 2021 |
| 10 | Ivanova I, Seledtsov V, Seledtsova G, Mamaev S, Potyemkin A, Seledtsov D, et al. Induction of antiidiotypic immune response with autologous T-cell vaccine in patients with multiple sclerosis. Bulletin of experimental biology and medicine. 2008;146:133-8 | December 2021 |
| 11 | Loftus B, Newsom B, Montgomery M, Von Gynz-Rekowski K, Riser M, Inman S, et al. Autologous attenuated T-cell vaccine (Tovaxin®) dose escalation in multiple sclerosis relapsing–remitting and secondary progressive patients nonresponsive to approved immunomodulatory therapies. Clinical Immunology. 2009;131(2):202-15. | December 2021 |
| 12 | Pender MP, Csurhes PA, Smith C, Douglas NL, Neller MA, Matthews KK, et al. Epstein-Barr virus–specific T cell therapy for progressive multiple sclerosis. JCI insight. 2018;3(22). | December 2021 |
| 13 | Zhang JZ, Rivera VM, Tejada-Simon MV, Yang D, Hong J, Li S, et al. T cell vaccination in multiple sclerosis: results of a preliminary study. Journal of neurology. 2002;249:212-8. | February 2022 |
| 14 | Zubizarreta I, Flórez-Grau G, Vila G, Cabezón R, España C, Andorra M, et al. Immune tolerance in multiple sclerosis and neuromyelitis optica with peptide-loaded tolerogenic dendritic cells in a phase 1b trial. Proc Natl Acad Sci U S A. 2019;116(17):8463-70. **PLUS** data obtained from the authors through mail in October 2021. | February 2022 |
| 15 | Bar-Or A, Vollmer T, Antel J, Arnold DL, Bodner CA, Campagnolo D, et al. Induction of Antigen-Specific Tolerance in Multiple Sclerosis After Immunization With DNA Encoding Myelin Basic Protein in a Randomized, Placebo-Controlled Phase 1/2 Trial. Archives of Neurology. 2007;64(10):1407-15. | February 2022 |
| 16 | Garren H, Robinson WH, Krasulová E, Havrdová E, Nadj C, Selmaj K, et al. Phase 2 trial of a DNA vaccine encoding myelin basic protein for multiple sclerosis. Annals of neurology. 2008;63(5):611-20 | February 2022 |
| 17 | Bourdette DN, Edmonds E, Smith C, Bowen J, Guttmann CR, Nagy Z, et al. A highly immunogenic trivalent T cell receptor peptide vaccine for multiple sclerosis. Multiple Sclerosis Journal. 2005;11(5):552-61. | February 2022 |
| 18 | Freedman M, Bar-Or A, Oger J, Traboulsee A, Patry D, Young C, et al. A phase III study evaluating the efficacy and safety of MBP8298 in secondary progressive MS. Neurology. 2011;77(16):1551-60. | February 2022 |
| 19 | Goodkin DE, Shulman M, Winkelhake J, Waubant E, Andersson P-B, Stewart T, et al. A phase I trial of solubilized DR2: MBP84-102 (AG284) in multiple sclerosis. Neurology. 2000;54(7):1414-20. | February 2022 |
| 20 | Kappos L, Comi G, Panitch H, Oger J, Antel J, Conlon P, et al. Induction of a non-encephalitogenic type 2 T helper-cell autoimmune response in multiple sclerosis after administration of an altered peptide ligand in a placebo-controlled, randomized phase II trial. Nature medicine. 2000;6(10):1176-82. | March 2022 |
| 21 | van Noort JM, Bsibsi M, Nacken PJ, Verbeek R, Venneker EH. Therapeutic intervention in multiple sclerosis with alpha B-crystallin: A randomized controlled phase IIa trial. PloS one. 2015;10(11):e0143366. | March 2022 |
| 22 | Yadav V, Bourdette DN, Bowen JD, Lynch SG, Mattson D, Preiningerova J, et al. Recombinant T-cell receptor ligand (RTL) for treatment of multiple sclerosis: a double-blind, placebo-controlled, phase 1, dose-escalation study. Autoimmune diseases. 2012;2012. | March 2022 |
| 23 | Walczak A, Siger M, Ciach A, Szczepanik M, Selmaj K. Transdermal application of myelin peptides in multiple sclerosis treatment. JAMA neurology. 2013;70(9):1105-9. | March 2022 |
| 24 | Warren K, Catz I, Ferenczi L, Krantz M. Intravenous synthetic peptide MBP8298 delayed disease progression in an HLA Class II‐defined cohort of patients with progressive multiple sclerosis: results of a 24‐month double‐blind placebo‐controlled clinical trial and 5 years of follow‐up treatment. European journal of neurology. 2006;13(8):887-95. | March 2022 |
| 25 | Karussis D, Shor H, Yachnin J, Lanxner N, Amiel M, Baruch K, et al. T cell vaccination benefits relapsing progressive multiple sclerosis patients: a randomized, double-blind clinical trial. PloS one. 2012;7(12):e50478. | March 2022 |
| 26 | Fox E, Wynn D, Cohan S, Rill D, McGuire D, Markowitz C. A randomized clinical trial of autologous T-cell therapy in multiple sclerosis: subset analysis and implications for trial design. Multiple Sclerosis Journal. 2012;18(6):843-52. | March 2022 |
| **Excluded studies** | | |
| **Study** | **Name of the study** | **Reason for exclusion** |
| 27 | https://clinicaltrials.gov/study/NCT02057159 | Study finished, but no published results |
| 28 | https://clinicaltrials.gov/study/NCT00079495 | Study finished, but no published results |
| 29 | https://clinicaltrials.gov/study/NCT02149706 | Study finished, but no published results |
| 30 | https://clinicaltrials.gov/study/NCT00869986 | Study finished, but no published results |
| 31 | https://clinicaltrials.gov/study/NCT01684761 | Study finished, but no published results |
| 32 | https://clinicaltrials.gov/study/NCT00228228 | Study finished, but no published results |
| 33 | https://clinicaltrials.gov/study/NCT00468611 | Terminated and no published results |
| 34 | https://ctv.veeva.com/study/a-study-for-patients-with-multiple-sclerosis | Terminated and no published results |
| 35 | https://clinicaltrials.gov/study/NCT00001781?term=NCT00001781&rank=1 | Terminated and no published results |
| 36 | https://clinicaltrials.gov/study/NCT02427776?term=NCT02427776&rank=1 | Terminated due to unexpected issues in the manufacturing process, before enrolment of the first patient |
| 37 | https://clinicaltrials.gov/study/NCT00595920?term=NCT00595920&rank=1&tab=results | Terminated due to financial constraints |
| 38 | https://www.clinicaltrialsregister.eu/ctr-search/search?query=eudract_number:2004-002571-16 | No published results |
| 39 | https://clinicaltrials.gov/study/NCT00220428 | No published results |
| 40 | https://clinicaltrial.be/en/details/72626?per_page=20&only_recruiting=0&only_eligible=0&only_active=0 | Ongoing study |
| 41 | https://www.clinicaltrialsregister.eu/ctr-search/trial/2016-002180-33/PL | Ongoing study |
| 42 | https://clinicaltrials.gov/study/NCT02618902?tab=results | Ongoing study |
| 43 | https://clinicaltrials.gov/study/NCT02903537 | Ongoing study |
| 44 | https://clinicaltrials.gov/study/NCT04530318 | Ongoing study |
| 45 | https://www.neurology.org/doi/10.1212/wnl.0b013e318233b240 | Duplicate study, Freedman et al, 2011 |
| 46 | https://clinicaltrials.gov/study/NCT00103974 | Duplicate study, Bar-Or et al, 2007. |
| 47 | https://clinicaltrials.gov/study/NCT02442557?term=NCT02442557&rank=1&tab=results | Duplicate study, van Noort, et al, 2015. |
| 48 | https://clinicaltrials.gov/study/NCT02442570?term=NCT02442570&rank=1&tab=results | Duplicate study, van Noort, et al, 2015. |
| 49 | https://clinicaltrials.gov/study/NCT01973491?tab=results | Duplicate study, Chataway, et al, 2018. |
| 50 | https://www.pnas.org/doi/10.1073/pnas.1820039116 | Duplicate study, already included, Zubizareta et al, 2019. |
| 51 | <https://clinicaltrials.gov/study/NCT00245622?tab=results> | Duplicate study, already included, Fox et al, 2012. |
| 52 | https://clinicaltrials.gov/study/NCT00411723?tab=results | Duplicate study (results presented in Yadav et al, 2012) |
| 53 | https://clinicaltrials.gov/study/NCT00587691?tab=results | Duplicate study (results presented in Zhang et al, 2002) |
| 54 | https://clinicaltrials.gov/study/NCT01448252?term=NCT01448252&rank=1&tab=results | Duplicate study (results presented in Karussis et al, 2012) |
| 55 | https://clinicaltrials.gov/study/NCT02283671?term=NCT02283671&rank=1&tab=results | Duplicate study (results presented in Zubizarreta et al, 2019) |
| 56 | https://www.webofscience.com/wos/woscc/full-record/WOS:000275274002589 | Abstract presented at the 62nd Annual Meeting of the American-Academy-of-Neurology (results presented later in Karussis et al, 2012) |
| 57 | https://link.springer.com/article/10.1007/s00415-007-3001-6 | Abstract presented at the conference (Garren et al, 2008) |
| 58 | <https://pubmed.ncbi.nlm.nih.gov/4123119/> | Ineligible outcomes |
| 59 | https://www.jni-journal.com/article/S0165-5728(10)00432-7/fulltext | Ineligible outcomes |
| 60 | https://pubmed.ncbi.nlm.nih.gov/9395124/ | Ineligible outcomes |
| 61 | https://www.nature.com/articles/nm1000_1167 | Insufficient patient number |
| 62 | https://pubmed.ncbi.nlm.nih.gov/11079539/ | Insufficient patient number |
| 63 | <https://pubmed.ncbi.nlm.nih.gov/8583237/> | Insufficient patient number |
| 64 | https://link.springer.com/article/10.1007/s00415-006-2001-2 | Abstract only presented at the conference |
| 65 | <https://plus.cobiss.net/cobiss/sr/en/bib/1025611189> | Abstract presented at the conference (Garren et al, 2008) |
| 66 | https://journals.sagepub.com/doi/abs/10.1177/1352458509106963 | Abstract presented at the conference (Garren et al, 2008) |
| 67 | https://link.springer.com/article/10.1007/s00415-006-2001-2 | Abstract presented at the conference (Garren et al, 2008) |
| 68 | https://www.webofscience.com/wos/woscc/full-record/WOS:000264527902154 | Abstract only presented at the [61st Annual Meeting of American Academy of Neurology](https://www.webofscience.com/wos/woscc/general-summary?queryJson=%5B%7B%22rowBoolean%22:null,%22rowField%22:%22CF%22,%22rowText%22:%2261st%20Annual%20Meeting%20of%20American-Academy-of-Neurology%22%7D%5D&eventMode=oneClickSearch); the authors were contacted and said these data were post-hoc analysis results of the previously published results in Garren et al, 2008. |
| 69 | Garren et al. Results from a phase 2b trial of a myelin basic protein encoding DNA vaccine for relapsing multiple sclerosis, 2008. | Abstract presented at the [60th Annual Meeting of American Academy of Neurology](https://www.webofscience.com/wos/woscc/general-summary?queryJson=%5B%7B%22rowBoolean%22:null,%22rowField%22:%22CF%22,%22rowText%22:%2261st%20Annual%20Meeting%20of%20American-Academy-of-Neurology%22%7D%5D&eventMode=oneClickSearch) (results published in Garren et al, 2008. ) |
| 70 | https://pubmed.ncbi.nlm.nih.gov/9184630/ | Ineligible outcomes |
| 71 | https://pubmed.ncbi.nlm.nih.gov/1541056/ | Insufficient patient number |
| 72 | https://www.webofscience.com/wos/woscc/full-record/WOS:000259675700138 | Abstract presented at the conference (results published in Garren et al, 2008. ) |
| 73 | <https://journals.sagepub.com/doi/abs/10.1191/1352458505ms1258xx> | Abstract presented at the conference (results published in Garren et al, 2008. ) |
| 74 | https://www.neurology.org/doi/10.1212/WNL.86.16_supplement.P2.159 | Abstract presented at the conference (results published in Zubizarreta et al, 2019. ) |
| 75 | https://www.neurology.org/doi/10.1212/WNL.92.15_supplement.S56.002 | Based on this abstract, we contacted authors and obtained additional information on the study Zubizarreta et al, 2019, this is included |
| 76 | https://www.isrctn.com/ISRCTN98373474 | No results posted (but results are presented in Freedman et al ,2011) |
| 77 | https://onlinelibrary.wiley.com/doi/abs/10.1002/ana.22219 | Ineligible outcomes |
| 78 | https://pubmed.ncbi.nlm.nih.gov/27239100/ | Ineligible outcomes |
| 79 | https://pubmed.ncbi.nlm.nih.gov/23246830/ | Ineligible outcomes |
| 80 | https://pubmed.ncbi.nlm.nih.gov/9125392/ | Ineligible outcomes |
| 81 | https://pubmed.ncbi.nlm.nih.gov/22222856/ | Ineligible study design (retrospective study) |
| 82 | https://journals.sagepub.com/doi/abs/10.1177/1352458516663067 | Ineligible study design (study protocol) |
| 83 | https://pubmed.ncbi.nlm.nih.gov/31501122/ | Ineligible study design (study protocol) |
| 84 | https://journals.sagepub.com/doi/abs/10.1177/1352458512461105?journalCode=msja | Insufficient patient number |
| 85 | https://pubmed.ncbi.nlm.nih.gov/23740901/ | Insufficient patient number |
| 86 | https://pubmed.ncbi.nlm.nih.gov/9345417/ | Insufficient patient number |
| 87 | https://www.neurology.org/doi/full/10.1212/NXI.0000000000000093 | Insufficient patient number |
| 88 | https://www.neurology.org/doi/10.1212/WNL.88.16_supplement.P2.330 | Abstract presented at the conferences (results published in Zubizarreta et al, 2019) |
